# The quality of life in neoadjuvant versus adjuvant therapy of esophageal cancer treatment trial (QUINTETT): Randomized parallel clinical superiority trial

**DOI:** 10.1111/1759-7714.14433

**Published:** 2022-05-24

**Authors:** Richard A. Malthaner, Edward Yu, Michael Sanatani, Debra Lewis, Andrew Warner, A. Rashid Dar, Brian P. Yaremko, Joel Bierer, David A. Palma, Dalilah Fortin, Richard I. Inculet, Eric Fréchette, Jacques Raphael, Stewart Gaede, Sara Kuruvilla, Jawaid Younus, Mark D. Vincent, George B. Rodrigues

**Affiliations:** ^1^ Department of Oncology, Divisions of Thoracic Surgery and Surgical Oncology, Schulich School of Medicine and Dentistry Western University London Ontario Canada; ^2^ Radiation Oncology, Schulich School of Medicine and Dentistry Western University London Ontario Canada; ^3^ Medical Oncology, Schulich School of Medicine and Dentistry Western University London Ontario Canada

**Keywords:** adjuvant, esophageal cancer, neoadjuvant, quality of life, randomized, trimodality

## Abstract

**Background:**

We compared the health‐related quality of life (HRQOL) in patients undergoing trimodality therapy for resectable stage I‐III esophageal cancer.

**Methods:**

A total of 96 patients were randomized to standard neoadjuvant cisplatin and 5‐fluorouracil chemotherapy plus radiotherapy (neoadjuvant) followed by surgical resection or adjuvant cisplatin, 5‐fluorouracil, and epirubicin chemotherapy with concurrent extended volume radiotherapy (adjuvant) following surgical resection.

**Results:**

There was no significant difference in the functional assessment of cancer therapy‐esophageal (FACT‐E) total scores between arms at 1 year (*p* = 0.759) with 36% versus 41% (neoadjuvant vs. adjuvant), respectively, showing an increase of ≥15 points compared to pre‐treatment (*p* = 0.638). The HRQOL was significantly inferior at 2 months in the neoadjuvant arm for FACT‐E, European Organization for Research and Treatment of Cancer quality of life questionnaire (EORTC QLQ‐OG25), and EuroQol 5‐D‐3 L in the dysphagia, reflux, pain, taste, and coughing domains (*p* < 0.05). Half of patients were able to complete the prescribed neoadjuvant arm chemotherapy without modification compared to only 14% in the adjuvant arm (*p* < 0.001). Chemotherapy related adverse events of grade ≥2 occurred significantly more frequently in the neoadjuvant arm (100% vs. 69%, *p* < 0.001). Surgery related adverse events of grade ≥2 were similar in both arms (72% vs. 86%, *p* = 0.107). There were no 30‐day mortalities and 2% vs. 10% 90‐day mortalities (*p* = 0.204). There were no significant differences in either overall survival (OS) (5‐year: 35% vs. 32%, *p* = 0.409) or disease‐free survival (DFS) (5‐year: 31% vs. 30%, *p* = 0.710).

**Conclusion:**

Trimodality therapy is challenging for patients with resectable esophageal cancer regardless of whether it is given before or after surgery. Newer and less toxic protocols are needed.

## Graphical abstract

This randomized trial assessed the health related quality of life in esophageal cancer patients undergoing neoadjuvant versus adjuvant trimodality therapy. Every patient experienced at least one adverse event. The quality of life was worse for patients undergoing neoadjuvant compared to adjuvant chemoradiation with no significant difference in either overall survival or disease‐free survival. More effective therapy is needed.

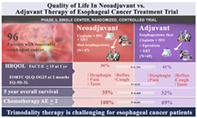

## INTRODUCTION

Esophageal cancer is a challenging disease entity that reduces a patient's health‐related quality‐of‐life (HRQOL) and is lethal in most cases.^1^ Although some patients can be cured, the treatment for esophageal cancer is protracted and diminishes HRQOL.^2^, ^3^ There are only a few studies assessing the HRQOL of patients undergoing multi‐modality therapy for esophageal cancer, but there are no reports evaluating the HRQOL of patients undergoing neoadjuvant compared to adjuvant chemoradiation in patients undergoing esophagectomy.^2^, ^4^, ^5^, ^6^

Epirubicin, cisplatin, and 5‐fluorouracil (ECF) along with extended field radiation that includes the anastomosis given in the adjuvant setting has been the standard of care at our center for several decades.^7^, ^8^, ^9^, ^10^ We set out to compare the HRQOL effects of standard neoadjuvant cisplatin and 5‐fluorouracil (5‐FU) chemotherapy plus radiotherapy (neoadjuvant) followed by surgical resection to adjuvant cisplatin, 5‐FU, and epirubicin chemotherapy with concurrent extended volume radiotherapy (adjuvant) following surgical resection for resectable esophageal carcinoma.

## OBJECTIVE

We hypothesized that both trimodality treatment options would provide similar survival results but may have different patient‐centered experiences with respect to HRQOL and adverse events.

## TRIAL DESIGN

The Quality of Life in Neoadjuvant vs. Adjuvant Therapy of Esophageal Cancer Treatment Trial (QUINTETT) was a single‐center, parallel‐group, centrally concealed, randomized superiority study. The Consolidated Standards of Reporting Trials (CONSORT) 2010 guideline was followed to report the details of this trial.^11^

## PARTICIPANTS

**FIGURE 1:**
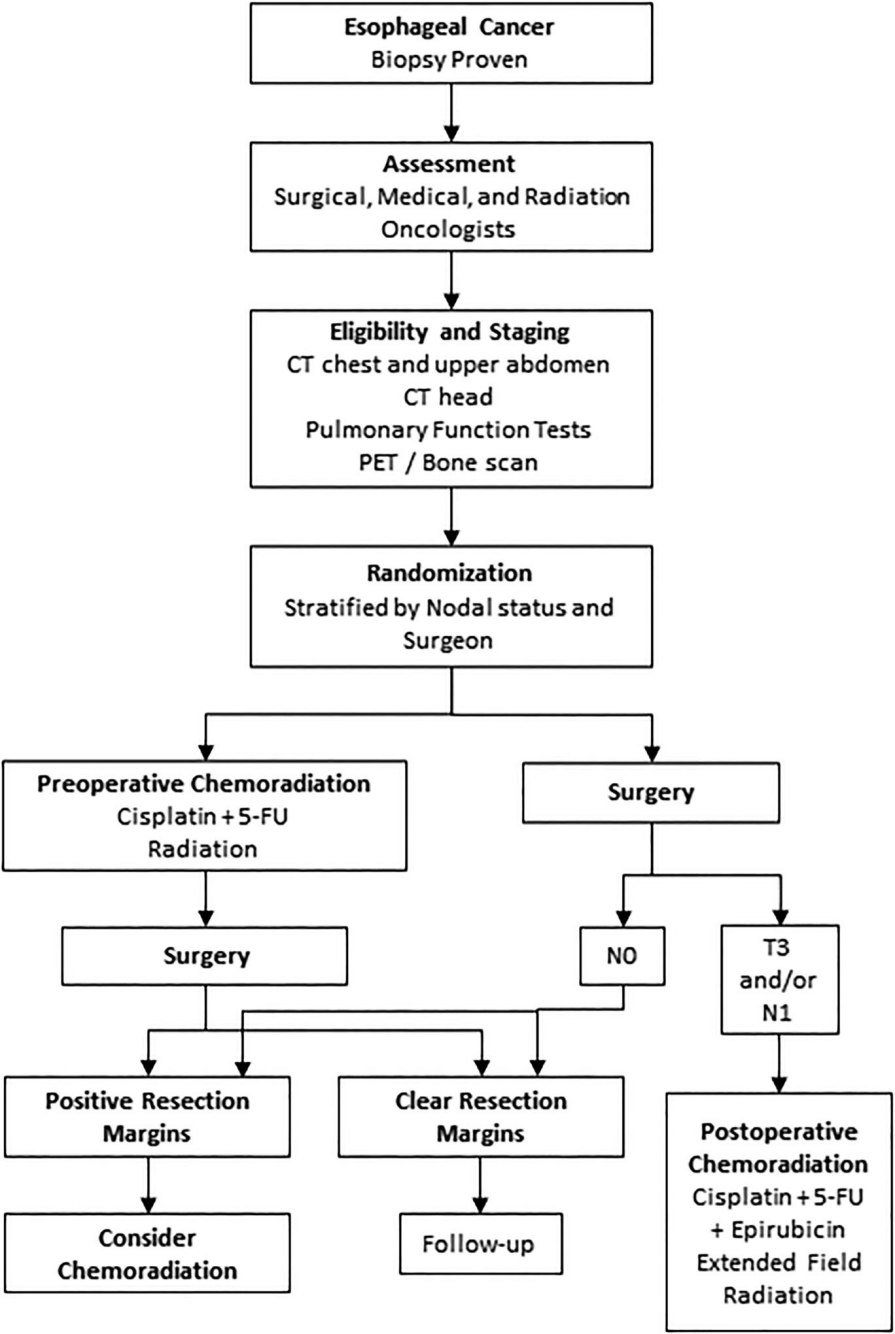
Study schema. CT, computed tomography; PET, positron emission tomography; 5‐FU, 5‐fluorouracil

The study population consisted of sequentially screened patients with stage I to III resectable cancer of the esophagus referred to our institution (Figure 1). The inclusion criteria were histologically documented squamous cell carcinomas or adenocarcinomas of the thoracic esophagus (>20 cm from the incisors) or gastroesophageal junction (<3 cm into the stomach) that were judged to be operable, resectable, and able to tolerate trimodality therapy by a surgical, medical, and radiation oncologist.

## STUDY SETTING

The study took place from April 2009 to November 2016 at the London Health Sciences Centre and London Regional Cancer Program. The study was completed and approved by our local ethics review board in accordance with the Declaration of Helsinki, Good Clinical Practice guideline.^12^ All included patients provided written informed consent. Pre‐treatment clinical and final pathologic staging was based on the American Joint Committee on Cancer (7th edition).^13^

## NEOADJUVANT THERAPY

In the neoadjuvant arm treatment began within 4 weeks of randomization and consisted of 2 cycles of induction cisplatin 25 mg/m^2^ × 4 days (=100 mg/m^2^) concurrent with 5‐FU 1000 mg/m^2^ per day for 96 hours continuous venous infusion for 2 cycles every 28 days with concurrent 50.4 Gy in 28 fractions of radiation therapy. Additional details are available in the protocol in Appendix S1.

## ADJUVANT THERAPY

The adjuvant arm consisted of surgical resection followed by 2 cycles of ECF chemotherapy and concurrent extended volume radiation treatment that included the anastomosis and began between 8‐ and 12‐weeks following surgery. This was chemotherapy alone for 2 cycles (epirubicin 50 mg/m^2^ and cisplatin 60 mg/m^2^ day 1, and 5‐FU by continuous venous infusion at 200 mg/m^2^ for 21 days) then cisplatin 60 mg/m^2^ day 1, and 5‐FU by continuous venous infusion at 200 mg/m^2^ for 21 days with concurrent 50.4 Gy radiation immediately afterward for 2 additional cycles as previously described.^7^, ^13^, ^14^ Adjuvant chemoradiation was only given to patients with pathologic T3 or N1 disease. Patients with positive radial margins were offered chemoradiation at the discretion of the oncologists. Additional details are available in the protocol in the online Appendix S1.

## PRIMARY OUTCOME

The primary endpoint was the tumor‐specific patient‐oriented HRQOL using the validated functional assessment of cancer therapy: esophagus (FACT‐E) at 1 year.^15^ It is a HRQOL subscale for patients with esophageal cancer used with the functional assessment of cancer therapy: general (FACT‐G) and provides a more multidimensional assessment of HRQOL than the European Organization for Research and Treatment of Cancer Quality of Life questionnaire (EORTC QLQ)‐C30/QLQ‐OG25.^16^, ^17^, ^18^, ^19^, ^20^ The a priori minimal clinically important difference was conservatively set at 15 indicating that a 15‐point increase in the FACT‐E score compared to baseline would correspond with a clinically meaningful positive effect on HRQOL for the patient.^21^, ^22^ Additional details are provided in the Appendix S1 online protocol.

## SECONDARY ENDPOINTS

Secondary endpoints included additional HRQOL assessments using the tumor‐specific EORTC QLQ‐OG 25.^23^, ^24^, ^25^ The EuroQoL 5‐Dimension 3‐Level (EQ‐5D‐3L) utility instrument^25^, ^26^ that has minimal clinically important differences estimated to be 0.06 for US‐index scores.^27^ Other outcomes included adverse events using the Common Terminology Criteria for Adverse Events (CTCAE), version 4.0, and overall survival (OS) and disease‐free survival (DFS), both calculated from date of randomization to date of recurrence (DFS only), date of death because of any cause, or date of last follow‐up, whichever occurs first.^37^

## SAMPLE SIZE

The sample size was estimated based on an improvement in the HRQOL as measured by the FACT‐E at 1 year of 15 points. Based on a two‐sample *t*‐test, using two‐sided testing, α = 0.05, power = 80% and standard deviation (SD) = 25; 48 patients would be required in each arm (96 total patients) after adjusting for 10% lost to follow‐up.^28^ The randomization was completed in a 1:1 allocation ratio, stratified by nodal status and surgeon according to a computer‐generated randomization list by an independent statistician to ensure concealment of the randomization process.^29^ There was no blinding.

## STATISTICAL METHODS

Descriptive statistics were generated for all patients and compared using the χ^2^ test, Fisher's exact test, two‐sample *t*‐test, Wilcoxon rank‐sum test, or paired *t*‐test as appropriate. Univariable Cox proportional hazards regression was performed for OS and DFS (Table S1). Kaplan–Meier estimates were generated for OS and DFS log‐rank test stratified by clinical nodal status. All analysis was intention‐to‐treat and performed using SAS version 9.4 software (SAS Institute) using two‐sided testing at the 0.05 significance level.

## RESULTS

### Participant flow

**FIGURE 2:**
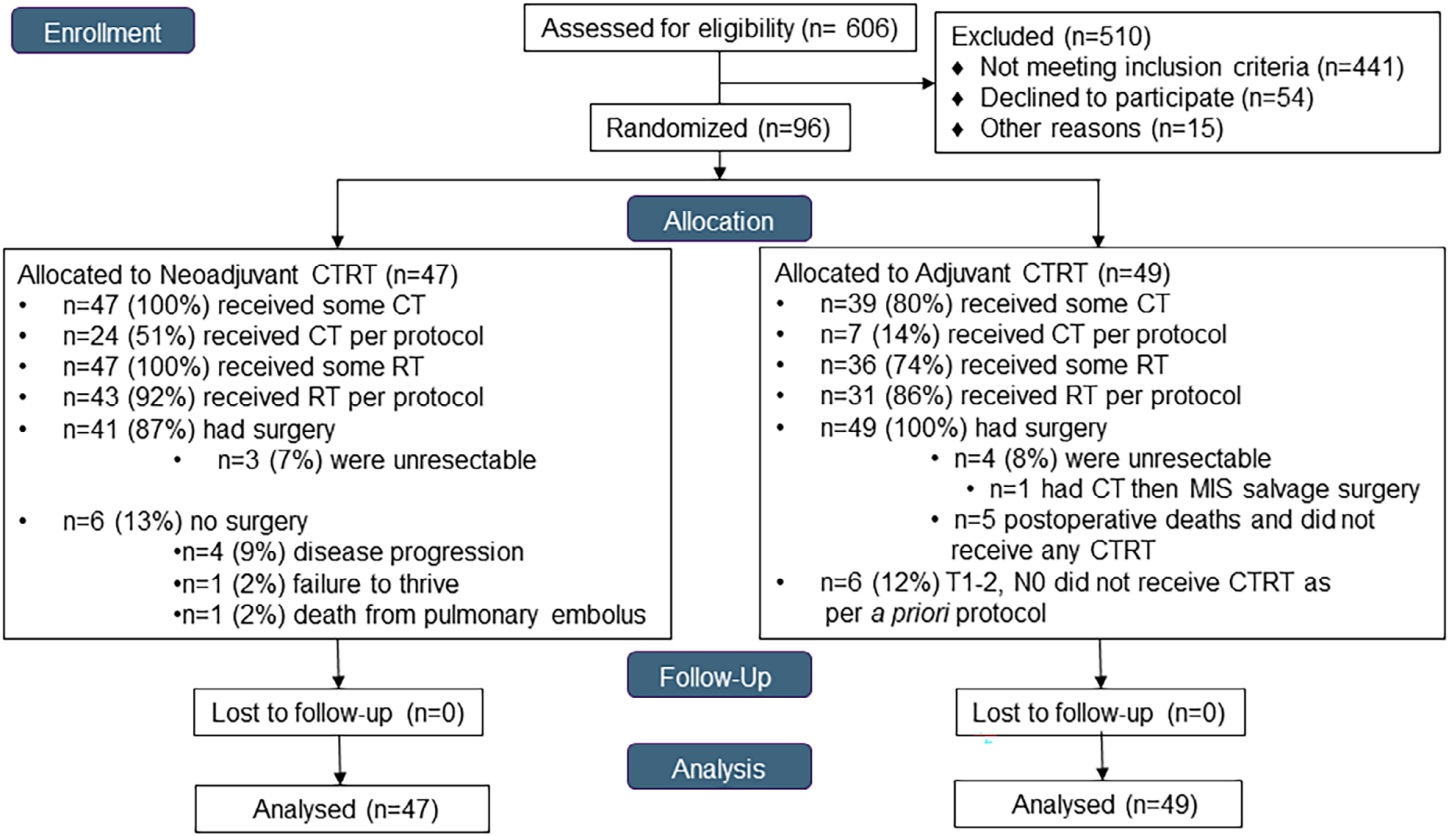
Consort diagram

A total of 606 consecutive patients referred to our institution. A total of 510 patients were excluded: 441 did not meet inclusion criteria; 54 declined to participate; and 15 for other reasons. Ninety‐six patients with stage I to III resectable esophageal cancer were ultimately randomized, 47 patients into neoadjuvant arm and 49 patients into adjuvant arm (Figure 2).

### Baseline data

Baseline characteristics are summarized in Table 1. Overall patients were well balanced. Most patients had adenocarcinomas of the distal esophagus/gastroesophageal junction (89% vs. 90%). The combined pathologic stage distribution was 0 9%; I 21%; II 31%; III 38%. A total of 92% of patients in the neoadjuvant arm received a percutaneous feeding tube and nutritional support compared to 96% of patients in the adjuvant arm. The median follow‐up was 7.6 years (95% confidence interval [CI], 6.5–9.5), 8.7 years (95% CI, 7.4–9.9) in the neoadjuvant arm and 7.6 years (95% CI, 5.1–11.8) in the adjuvant arm, and no patient was lost to follow‐up.

**TABLE 1:** Baseline patient characteristics for all patients and stratified by treatment arm (*n* = 96)

Characteristic	Arm 1: neoadjuvant CRT (*n* = 47)	Arm 2: adjuvant CRT (*n* = 49)	*p*‐value
Age, mean ± SD, median (IQR), y	63.1 ± 7.7 63.1 (57.6, 68.3)	65.6 ± 8.0 67.0 (60.2, 70.5)	0.112
Gender, *n* (%)			
Male	41 (87.2)	37 (75.5)	0.141
Female	6 (12.8)	12 (24.5)	
Clinical stage, *n* (%)			
T1N0	4 (8.5)	5 (10.2)	–
T1N1	0 (0)	2 (4.1)	
T2N0	13 (27.7)	10 (20.4)	
T2N1	2 (4.3)	2 (4.1)	
T3N0	12 (25.5)	17 (34.7)	
T3N1	14 (29.8)	13 (26.5)	
TXN0	1 (2.1)	0 (0)	
TXN1	1 (2.1)	0 (0)	
Histology, *n* (%)			
Adenocarcinoma	42 (89.4)	44 (89.8)	0.191
Squamous	2 (4.3)	5 (10.2)	
Unknown	3 (6.4)	0 (0)	
SUV_Max_, mean ± SD, median (IQR)	9.6 ± 6.2 8.9 (4.9, 12.2)	9.0 ± 6.9 6.8 (4.5, 10.4)	0.343
Median follow‐up (y),^a^ median (95% CI)	8.70 (7.36, 9.93)	7.61 (5.13, 11.78)	0.869

Abbreviations: CI, confidence interval; CRT, chemoradiotherapy; IQR, interquartile range; SUV_Max_, maximum standardized uptake values.

^a^ Reverse Kaplan–Meier method (calculated from date of randomization).

### Outcomes

HRQOL

**FIGURE 3:**
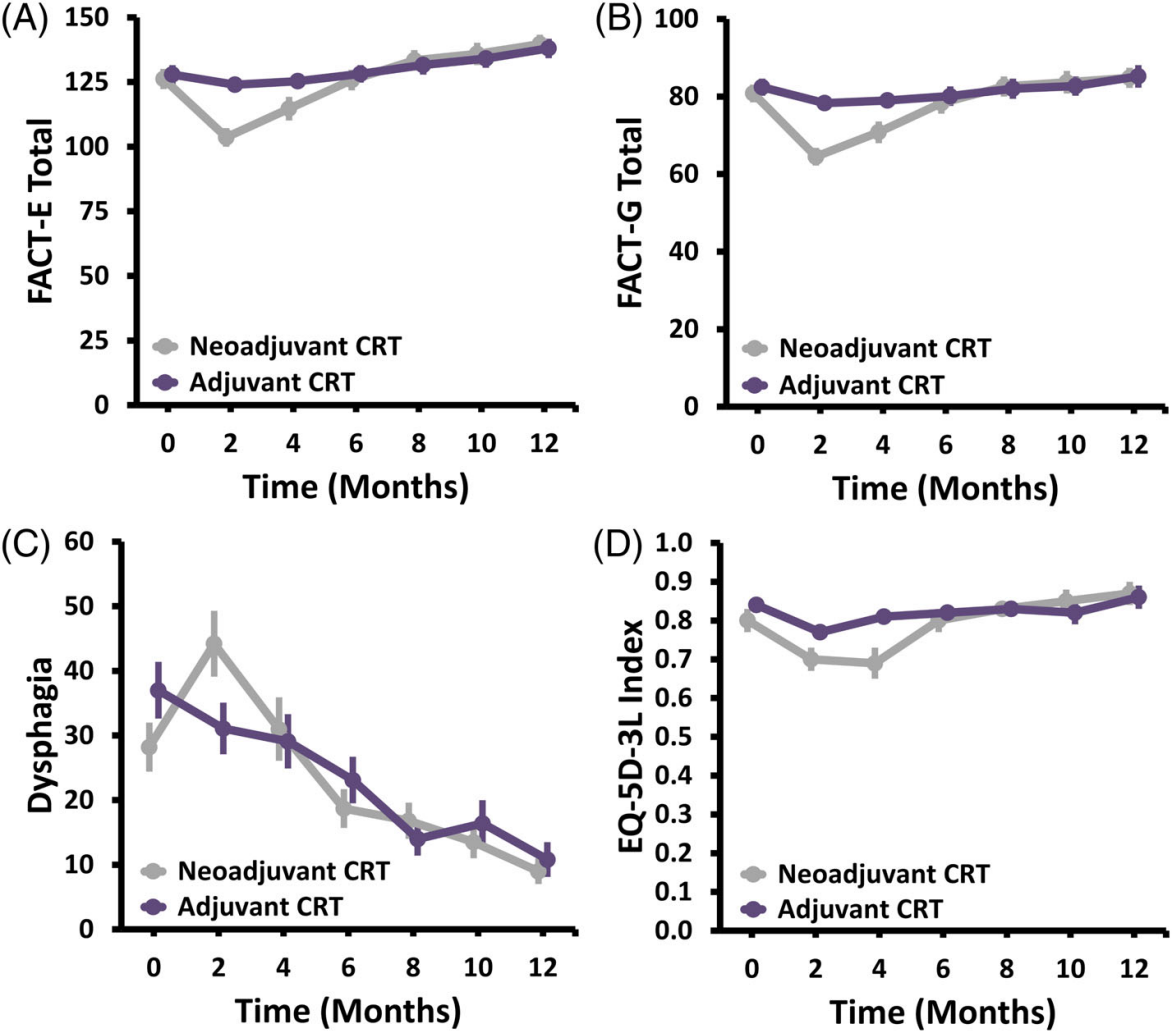
(a–d). Health‐related quality‐of‐life over time stratified by treatment arm for (a) FACT‐E total, (b) FACT‐G total, (c) EORTC QLQ‐OG25 dysphagia, and (d) EQ‐5D‐3L index. Abbreviations: FACT‐E – Functional Assessment of Cancer Therapy: Esophagus; FACT‐G – Functional Assessment of Cancer Therapy: General; EORTC QLQ‐OG25 – European Organization for Research and Treatment of Cancer Quality of Life Questionnaire: Oesophago‐Gastric 25; EQ‐5D‐3L – EuroQol 5‐Dimension 3‐Level

At baseline there were no statistically significant differences in HRQOL. At 1‐year follow‐up, the mean ± SD FACT‐E score was 139.7 ± 20.0 in the neoadjuvant arm compared to 138.0 ± 21.9 in the adjuvant arm with a mean difference of 1.7 (*p* = 0.759) (Table 2 and Figure 3(a)). Both arms improved significantly compared to baseline, but were below the a priori minimal clinically important difference threshold of 15. The FACT‐E score improved by ≥15 points in 11 (36%) of the patients in the neoadjuvant arm and 14 (41%) in the adjuvant arm (*p* = 0.638). An improvement of more than 15 points is considered clinically significant for patients. Overall, just over one‐third of patients receiving neoadjuvant therapy had a clinically significant improvement in their HRQOL compared to less than one half of patient receiving adjuvant chemoradiation. FACT‐E scores over time stratified by arm are shown in Figure 3(a). There was a clinically and statistically significant greater decrease in the HRQOL in the neoadjuvant arm at 2 months (−21.9 ± 28.4) compared to the adjuvant arm (−5.1 ± 26.1, *p* = 0.007). The Trial Outcome Index (defined as the sum of FACT‐G physical and functional well‐being and esophagus cancer subscale) also showed a significantly larger decrease for the neoadjuvant arm at 2 months (*p* = 0.008) and a significant improvement at 1‐year for the neoadjuvant arm (11.8 ± 26.3, *p* = 0.019), but not for the adjuvant arm (6.9 ± 25.0, *p* = 0.116). Similarly, the esophageal cancer subscale also decreased more for the neoadjuvant arm at 2 months and had a significant improvement at 1‐year for both arms. Although the magnitude of improvement at 1‐year for FACT‐E, Trial Outcome Index, and esophagus cancer subscale was greater for the neoadjuvant arm, this was not statistically significant.

Our study also found FACT‐G physical well‐being was significantly worse for the neoadjuvant arm at 2 months, translating to a decrease of −8.2 ± 8.3 compared to baseline for the neoadjuvant arm versus −2.0 ± 6.3 for the adjuvant arm, however, both returned to similar baseline values by 1 year and were not significantly different (neoadjuvant: *p* = 0.330; adjuvant: *p* = 0.550) (Figure 3(b) and Table 2).

Similar trends observed for FACT‐G and FACT‐E were also observed for EORTC QLQ‐OG25 and the EQ‐5D‐3L (Table 3 and Figure 3(d)). The EORTC QLQ‐C30 produces scores on 15 subscales (range for each, 0–100), with higher scores indicating better outcomes on global quality of life/health and functioning subscales and worse outcomes on symptom scales and for financial problems. For EORTC QLQ‐OG25, mean ± SD dysphagia was both clinically and statistically significantly worse at 2 months and resolved by 6 months in the neoadjuvant arm and significantly improved at 1 year for both arms (Figure 3(c) and Table 3).

**FIGURE 4:**
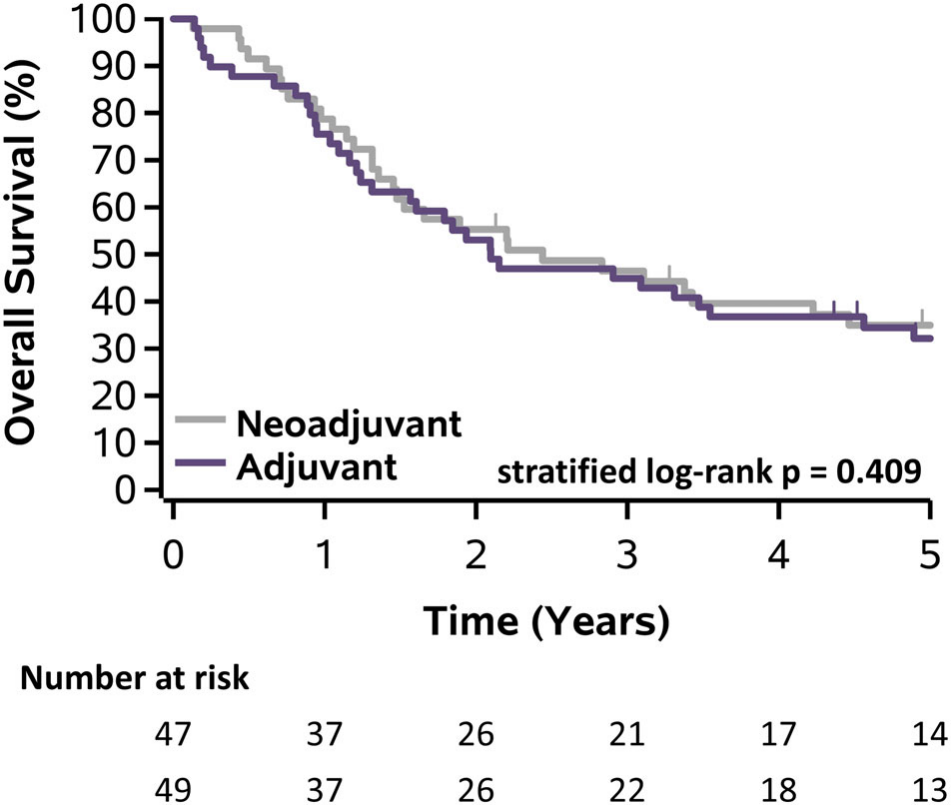
Kaplan‐Meier plot of overall survival stratified by treatment arm

For EQ‐5D‐3L, health state decreased significantly for both arms at 2 and 4 months, to a greater extent in the neoadjuvant arm, but was not significantly different and both arms returned to baseline by 6 months. The EQ‐5D‐3L Index (based on quality‐adjusted‐life‐years) was 0.80 ± 0.18 in the neoadjuvant arm and 0.84 ± 0.15 in the adjuvant arm at baseline, clinically and statistically decreased at 2 and 4 months for the neoadjuvant arm, and decreased at 2 months for adjuvant arm during the 6 months of trimodality therapy but returned to baseline values also by 6 months (Figure 3(d) and Table 3).

### Treatment

#### Chemoradiotherapy

A total of 49% of patients in the neoadjuvant arm required the chemotherapy to be modified or stopped compared to 57% in the adjuvant arm (*p* = 0.421) (Table 4). A total of 51% of patients were able to complete the prescribed neoadjuvant chemotherapy without modification compared to only 14% in the adjuvant arm (*p* < 0.001). Thirteen patients (26%) in the adjuvant arm did not receive the allocated chemotherapy and radiation (Table 4). All 47 patients in the neoadjuvant arm received some radiation, of which 43 (92%) patients completed the prescribed dose compared to 36 (74%) patients and 31 (86%) patients, respectively, in the adjuvant arm. Two patients (4%) in the neoadjuvant and 3 patients (6%) in the adjuvant received additional radiotherapy (Table 4).

**TABLE 2:** Summary of quality of life endpoints for all patients and stratified by treatment arm and follow‐up visit (*n* = 96)

Variable	Follow‐up visit								
	Baseline	2 month	4 month	6 month	1 year	2 month vs. baseline	4 month vs. baseline	6 month vs. baseline	1 year vs. baseline
FACT‐G Physical, mean ± SD									
Arm 1: neoadjuvant CRT	22.2 ± 6.2	14.1 ± 7.7	18.6 ± 6.9	21.0 ± 4.9	23.7 ± 3.9	−8.2 ± 8.3	−3.8 ± 6.9	−1.7 ± 6.1	1.2 ± 6.9
*p*‐value	—	—	—	—	—	<0.001*	0.002*	0.109	0.330
Arm 2: adjuvant CRT	23.3 ± 5.2	21.5 ± 3.8	21.1 ± 4.1	21.3 ± 5.7	22.9 ± 6.0	−2.0 ± 6.3	−2.6 ± 5.2	−2.2 ± 7.0	−0.7 ± 6.8
*p*‐value	—	—	—	—	—	0.042*	0.003*	0.057	0.550
Overall *p*‐value^a^	0.361	<0.001*	0.059	0.822	0.540	<0.001*	0.375	0.747	0.261
FACT‐G social, mean ± SD									
Arm 1: neoadjuvant CRT	23.7 ± 4.2	22.5 ± 5.3	22.9 ± 4.8	22.7 ± 6.2	22.7 ± 4.3	−1.0 ± 3.9	−0.5 ± 3.0	−0.6 ± 5.0	−0.8 ± 3.7
*p*‐value	—	—	—	—	—	0.124	0.272	0.452	0.234
Arm 2: adjuvant CRT	24.0 ± 4.8	23.8 ± 4.4	23.3 ± 5.0	23.0 ± 4.6	23.5 ± 4.8	−0.3 ± 3.3	−0.7 ± 4.1	−0.8 ± 3.7	−0.5 ± 4.8
*p*‐value	—	—	—	—	—	0.577	0.295	0.172	0.533
Overall *p*‐value^a^	0.804	0.211	0.734	0.770	0.475	0.380	0.874	0.875	0.784
FACT‐G emotional, mean ± SD									
Arm 1: neoadjuvant CRT	17.0 ± 4.4	17.1 ± 3.6	16.7 ± 4.7	19.6 ± 3.8	19.9 ± 4.1	0.2 ± 4.1	−0.1 ± 4.4	2.6 ± 4.3	3.2 ± 5.8
*p*‐value	—	—	—	—	—	0.756	0.883	0.001*	0.005*
Arm 2: adjuvant CRT	17.4 ± 4.7	18.2 ± 4.5	18.9 ± 4.0	19.2 ± 4.0	20.1 ± 4.1	1.0 ± 4.3	1.7 ± 4.9	2.1 ± 4.2	3.3 ± 5.3
*p*‐value	—	—	—	—	—	0.133	0.027*	0.004*	<0.001*
Overall *p*‐value^a^	0.661	0.221	0.030*	0.681	0.833	0.393	0.080	0.576	0.907
FACT‐G functional, mean ± SD									
Arm 1: neoadjuvant CRT	17.9 ± 6.4	10.8 ± 6.1	12.5 ± 7.1	15.1 ± 7.1	18.6 ± 6.2	−6.4 ± 6.9	−4.9 ± 7.3	−2.3 ± 7.9	1.5 ± 7.7
*p*‐value	—	—	—	—	—	<0.001*	<0.001*	0.088	0.294
Arm 2: adjuvant CRT	17.7 ± 7.1	14.7 ± 5.1	15.7 ± 5.8	16.6 ± 6.3	18.7 ± 6.5	−3.4 ± 6.9	−2.7 ± 5.9	−1.4 ± 6.1	1.1 ± 7.9
*p*‐value	—	—	—	—	—	0.002*	0.006*	0.172	0.428
Overall *p*‐value^a^	0.913	0.002*	0.028*	0.361	0.953	0.049*	0.132	0.550	0.839
FACT‐G total score^b^, mean ± SD									
Arm 1: neoadjuvant CRT	80.8 ± 15.4	64.5 ± 14.4	70.8 ± 17.2	78.3 ± 15.9	84.8 ± 14.7	−15.4 ± 16.9	−9.4 ± 15.0	−2.1 ± 17.7	5.1 ± 20.1
*p*‐value	—	—	—	—	—	<0.001*	<0.001*	0.494	0.171
Arm 2: adjuvant CRT	82.4 ± 15.2	78.3 ± 12.6	79.0 ± 13.1	80.1 ± 15.8	85.2 ± 16.8	−4.6 ± 14.1	−4.2 ± 13.0	−2.3 ± 15.3	3.2 ± 16.9
*p*‐value	—	—	—	—	—	0.035*	0.044*	0.353	0.278
Overall *p*‐value^a^	0.618	<0.001*	0.019*	0.641	0.927	0.002*	0.102	0.957	0.687
Esophagus cancer subscale, mean ± SD									
Arm 1: neoadjuvant CRT	45.5 ± 13.2	39.1 ± 10.3	44.0 ± 13.5	47.4 ± 9.7	54.8 ± 7.1	−6.5 ± 14.6	−1.7 ± 15.9	1.7 ± 14.3	9.0 ± 15.3
*p*‐value	—	—	—	—	—	0.009*	0.515	0.496	0.003*
Arm 2: adjuvant CRT	45.5 ± 12.4	45.6 ± 10.5	46.3 ± 9.8	48.1 ± 9.0	52.8 ± 8.0	−0.5 ± 14.9	0.0 ± 15.0	2.8 ± 15.0	6.5 ± 14.2
*p*‐value	—	—	—	—	—	0.816	0.991	0.251	0.011*
Overall *p*‐value^a^	1.00	0.006*	0.390	0.755	0.297	0.070	0.631	0.748	0.495
Trial outcome index^c^, mean ± SD									
Arm 1: neoadjuvant CRT	85.5 ± 22.4	64.0 ± 21.2	75.1 ± 24.8	83.6 ± 18.8	97.1 ± 15.3	−21.1 ± 25.9	−10.4 ± 26.0	−2.4 ± 23.9	11.8 ± 26.3
*p*‐value	—	—	—	—	—	<0.001*	0.018*	0.563	0.019*
Arm 2: adjuvant CRT	86.5 ± 22.1	81.9 ± 16.3	83.1 ± 15.2	86.0 ± 17.5	94.5 ± 16.6	−5.9 ± 25.1	−5.2 ± 22.2	−0.8 ± 25.0	6.9 ± 25.0
*p*‐value	—	—	—	—	—	0.129	0.134	0.848	0.116
Overall *p*‐value^a^	0.839	<0.001*	0.090	0.572	0.508	0.008*	0.344	0.779	0.450
FACT‐E total score^d^, mean ± SD									
Arm 1: neoadjuvant CRT	126.2 ± 25.4	103.6 ± 22.2	114.7 ± 28.6	125.8 ± 23.4	139.7 ± 20.0	−21.9 ± 28.4	−11.1 ± 28.4	−0.4 ± 28.7	14.1 ± 31.1
*p*‐value	—	—	—	—	—	<0.001*	0.021*	0.934	0.017*
Arm 2: adjuvant CRT	127.9 ± 25.5	124.0 ± 19.5	125.3 ± 18.4	128.2 ± 21.7	138.0 ± 21.9	−5.1 ± 26.1	−4.2 ± 23.9	0.5 ± 27.4	9.7 ± 27.1
*p*‐value	—	—	—	—	—	0.199	0.264	0.912	0.045*
Overall *p*‐value^a^	0.764	<0.001*	0.056	0.648	0.759	0.007*	0.246	0.892	0.548
FACT‐E total score improvement ≥15 points, *n* (%)									
Arm 1: neoadjuvant CRT	—	—	—	—	—	—	—	—	11 (35.5)
Arm 2: adjuvant CRT	—	—	—	—	—	—	—	—	14 (41.2)
Overall *p*‐value	—	—	—	—	—	—	—	—	0.638
FACT‐E total score improvement ≥9 points, *n* (%)									
Arm 1: neoadjuvant CRT	—	—	—	—	—	—	—	—	15 (48.4)
Arm 2: adjuvant CRT	—	—	—	—	—	—	—	—	18 (52.9)
Overall *p*‐value	—	—	—	—	—	—	—	—	0.714

Abbreviation: FACT‐G/E, functional assessment of cancer therapy: general/esophagus.

^a^ Reported from two‐sample *t*‐test.

^b^ Defined as sum of FACT‐G physical, social, emotional and functional well‐being.

^c^ Defined as sum of FACT‐G physical and functional well‐being and esophagus cancer subscale.

^d^ Defined as sum of FACT‐G physical, social, emotional and functional well‐being and esophagus cancer subscale.

* *p*‐values <0.05.

#### Surgery

Most patients underwent a minimally invasive esophagectomy with a laparoscopic transhiatal left neck approach (78% in the neoadjuvant arm and 82% in the adjuvant arm) (Table 5). The stomach was used for every conduit reconstruction. All 49 patients in the adjuvant arm underwent surgery, of which 4 (8%) patients were found to be unresectable compared to 41 (87%) patients in the neoadjuvant arm, of which 2 (5%) patients were found to be unresectable (Table 5). Only 2 (5%) patients in the neoadjuvant arm had positive margins, one proximal and one radial. In the adjuvant arm, 20 (44%) patients had positive margins, all 20 patients involving the radial margin and only 1 patient having a positive distal margin. A total of 92% of patients in the neoadjuvant arm received a feeding tube compared to 96% in the adjuvant arm. At 1‐month post‐discharge from surgery, 32% of patients in the neoadjuvant arm still had their feeding tube compared to only 10% in the adjuvant arm (*p* = 0.009). Six patients in the neoadjuvant arm did not undergo allocated surgical resection for the following reasons: spinal metastases (*n* = 1); peritoneal metastases (*n* = 1); disease progression during induction chemotherapy (*n* = 1); massive pulmonary embolus (*n* = 1); and sever cerebral vascular event (*n* = 1) (Figure 2).

**TABLE 3:** Summary of quality of life endpoints for all patients and stratified by treatment arm and follow‐up visit (*n* = 96)

Variable	Follow‐up visit								
	Baseline	2 month	4 month	6 month	1 year	2 month vs. baseline	4 month vs. baseline	6 month vs. baseline	1 year vs. baseline
EORTC OG25 dysphagia, mean ± SD									
Arm 1: neoadjuvant CRT	28.2 ± 25.2	44.2 ± 31.6	31.0 ± 30.4	18.7 ± 18.0	9.0 ± 11.3	17.1 ± 35.7	3.5 ± 34.3	−7.6 ± 28.5	−16.1 ± 29.7
*p*‐value	–	–	–	–	–	0.005*	0.532	0.123	0.005*
Arm 2: adjuvant CRT	37.0 ± 30.5	31.1 ± 26.4	29.1 ± 27.0	23.1 ± 22.6	10.8 ± 15.8	−5.3 ± 36.9	−6.6 ± 35.9	−13.9 ± 37.4	−24.8 ± 35.5
*p*‐value	–	–	–	–	–	0.345	0.239	0.024*	<0.001*
Overall *p*‐value^a^	0.133	0.045*	0.770	0.360	0.592	0.006*	0.202	0.414	0.286
EORTC OG25 eating, mean ± SD									
Arm 1: neoadjuvant CRT	38.1 ± 30.7	47.0 ± 25.9	40.7 ± 30.0	33.0 ± 21.1	21.7 ± 13.4	9.1 ± 33.8	2.8 ± 38.3	−4.8 ± 33.9	−14.6 ± 38.0
*p*‐value	–	–	–	–	–	0.103	0.654	0.411	0.041*
Arm 2: adjuvant CRT	42.9 ± 30.6	39.8 ± 24.5	36.7 ± 23.7	32.7 ± 24.2	25.1 ± 19.4	−0.6 ± 32.4	−4.0 ± 33.0	−9.7 ± 32.7	−15.7 ± 33.7
*p*‐value	–	–	–	–	–	0.902	0.441	0.069	0.011*
Overall *p*‐value^a^	0.460	0.200	0.510	0.957	0.415	0.188	0.402	0.526	0.906
EORTC OG25 reflux, mean ± SD									
Arm 1: neoadjuvant CRT	22.1 ± 24.0	31.6 ± 25.3	25.0 ± 30.2	20.0 ± 24.5	25.8 ± 21.0	8.3 ± 21.1	1.8 ± 25.3	−4.3 ± 34.1	0.0 ± 30.7
*p*‐value	–	–	–	–	–	0.020*	0.672	0.463	1.00
Arm 2: adjuvant CRT	24.3 ± 30.0	19.7 ± 21.9	20.6 ± 19.4	16.3 ± 18.7	16.2 ± 18.1	−1.9 ± 27.2	0.4 ± 25.6	−4.6 ± 25.9	−4.9 ± 31.4
*p*‐value	–	–	–	–	–	0.646	0.921	0.269	0.369
Overall *p*‐value^a^	0.697	0.027*	0.450	0.464	0.054	0.059	0.813	0.967	0.528
EORTC OG25 pain, mean ± SD									
Arm 1: neoadjuvant CRT	23.1 ± 26.4	34.0 ± 26.3	30.6 ± 27.7	21.2 ± 16.3	17.2 ± 13.9	9.6 ± 26.0	7.9 ± 29.3	−2.6 ± 29.9	−7.3 ± 33.8
*p*‐value	–	–	–	–	–	0.027*	0.111	0.608	0.241
Arm 2: adjuvant CRT	24.5 ± 19.6	20.6 ± 17.0	17.1 ± 17.5	17.1 ± 18.1	15.7 ± 16.9	−4.2 ± 21.6	−8.1 ± 21.3	−9.0 ± 22.0	−11.8 ± 27.1
*p*‐value	–	–	–	–	–	0.208	0.018*	0.014*	0.017
Overall *p*‐value^a^	0.774	0.009*	0.013*	0.305	0.693	0.011*	0.008*	0.305	0.558
EORTC OG25 anxiety, mean ± SD									
Arm 1: neoadjuvant CRT	46.3 ± 23.9	46.7 ± 26.4	46.2 ± 25.4	38.1 ± 23.5	25.1 ± 18.6	1.7 ± 21.6	0.6 ± 19.5	−5.7 ± 24.5	−20.4 ± 25.7
*p*‐value	–	–	–	–	–	0.623	0.854	0.176	<0.001*
Arm 2: adjuvant CRT	47.0 ± 26.3	44.7 ± 23.2	41.8 ± 21.5	39.4 ± 24.6	30.7 ± 23.9	−0.5 ± 23.5	−2.4 ± 27.3	−6.4 ± 27.6	−17.3 ± 31.4
*p*‐value	–	–	–	–	–	0.887	0.575	0.151	0.003*
Overall *p*‐value^a^	0.889	0.713	0.408	0.810	0.291	0.655	0.575	0.911	0.663
EORTC OG25 dry mouth, mean ± SD									
Arm 1: neoadjuvant CRT	27.9 ± 34.8	38.5 ± 34.7	28.1 ± 29.5	17.1 ± 26.0	15.1 ± 25.6	12.0 ± 37.1	1.8 ± 34.6	−7.6 ± 32.4	−11.8 ± 33.9
*p*‐value	–	–	–	–	–	0.051	0.757	0.174	0.062
Arm 2: adjuvant CRT	24.3 ± 32.1	29.5 ± 26.1	27.8 ± 25.4	30.8 ± 25.5	21.6 ± 23.0	6.1 ± 33.9	5.6 ± 33.7	8.3 ± 39.8	−2.0 ± 38.4
*p*‐value	–	–	–	–	–	0.243	0.291	0.193	0.768
Overall *p*‐value^a^	0.611	0.194	0.963	0.024*	0.287	0.453	0.620	0.060	0.276
EORTC OG25 taste, mean ± SD									
Arm 1: neoadjuvant CRT	17.8 ± 29.4	43.6 ± 39.8	30.7 ± 31.4	27.6 ± 33.8	18.3 ± 24.1	27.4 ± 45.2	15.8 ± 38.5	12.4 ± 39.7	2.2 ± 36.4
*p*‐value	–	–	–	–	–	<0.001*	0.016*	0.074	0.745
Arm 2: adjuvant CRT	11.1 ± 26.9	14.4 ± 19.6	28.6 ± 31.7	29.2 ± 35.6	15.7 ± 24.9	3.0 ± 35.8	18.3 ± 41.8	18.3 ± 44.0	8.8 ± 35.1
*p*‐value	–	–	–	–	–	0.578	0.007*	0.012*	0.152
Overall *p*‐value^a^	0.261	<0.001*	0.764	0.848	0.672	0.009*	0.785	0.540	0.456
EORTC OG25 body image, mean ± SD									
Arm 1: neoadjuvant CRT	17.8 ± 32.0	29.1 ± 36.0	32.5 ± 34.2	25.7 ± 30.3	17.2 ± 25.6	12.0 ± 40.8	16.7 ± 40.8	10.5 ± 28.9	1.1 ± 34.9
*p*‐value	–	–	–	–	–	0.075	0.016*	0.039*	0.865
Arm 2: adjuvant CRT	12.5 ± 25.4	22.0 ± 25.9	21.4 ± 24.2	26.7 ± 34.8	17.6 ± 28.7	11.4 ± 27.8	11.9 ± 30.2	16.7 ± 36.2	9.8 ± 32.3
*p*‐value	–	–	–	–	–	0.010*	0.014*	0.006*	0.086
Overall *p*‐value^a^	0.385	0.312	0.104	0.900	0.948	0.938	0.558	0.413	0.301
EORTC OG25 swallowing, mean ± SD									
Arm 1: neoadjuvant CRT	15.5 ± 29.4	20.5 ± 31.2	11.4 ± 24.8	8.6 ± 18.7	3.2 ± 10.0	3.4 ± 35.7	−6.1 ± 28.8	−6.7 ± 30.0	−11.8 ± 31.7
*p*‐value	–	–	–	–	–	0.553	0.198	0.198	0.046*
Arm 2: adjuvant CRT	11.1 ± 21.0	10.6 ± 22.5	9.5 ± 21.2	14.2 ± 27.1	6.9 ± 21.4	0.0 ± 28.8	−0.8 ± 25.0	3.3 ± 24.8	−2.9 ± 27.7
*p*‐value	–	–	–	–	–	1.00	0.838	0.401	0.540
Overall *p*‐value^a^	0.420	0.105	0.718	0.297	0.377	0.635	0.381	0.124	0.235
EORTC OG25 choking, mean ± SD									
Arm 1: neoadjuvant CRT	14.0 ± 22.1	15.4 ± 21.4	17.5 ± 24.2	10.5 ± 17.7	12.9 ± 16.5	2.6 ± 30.0	4.4 ± 25.9	−3.8 ± 27.7	0.0 ± 22.8
*p*‐value	–	–	–	–	–	0.597	0.303	0.422	1.00
Arm 2: adjuvant CRT	18.8 ± 25.6	18.9 ± 24.3	15.9 ± 24.7	18.3 ± 22.6	5.9 ± 15.3	2.3 ± 34.0	0.8 ± 31.7	2.5 ± 28.6	−10.8 ± 28.1
*p*‐value	–	–	–	–	–	0.660	0.872	0.584	0.032*
Overall *p*‐value^a^	0.341	0.481	0.761	0.096	0.081	0.967	0.579	0.337	0.093
EORTC OG25 coughing, mean ± SD									
Arm 1: neoadjuvant CRT	26.4 ± 27.8	26.5 ± 21.9	37.7 ± 30.2	33.3 ± 33.3	25.8 ± 25.4	5.1 ± 27.1	16.7 ± 32.7	13.3 ± 31.5	4.3 ± 29.5
*p*‐value	–	–	–	–	–	0.244	0.003*	0.017*	0.423
Arm 2: adjuvant CRT	30.6 ± 29.8	37.9 ± 25.5	36.5 ± 24.2	36.7 ± 27.0	28.4 ± 29.7	9.1 ± 36.2	9.5 ± 38.5	8.3 ± 33.5	−1.0 ± 39.8
*p*‐value	–	–	–	–	–	0.103	0.116	0.124	0.887
Overall *p*‐value^a^	0.489	0.032*	0.845	0.639	0.703	0.571	0.372	0.508	0.543
EORTC OG25 speech, mean ± SD									
Arm 1: neoadjuvant CRT	5.4 ± 14.4	12.0 ± 25.9	17.5 ± 27.7	15.2 ± 29.5	7.5 ± 20.6	6.0 ± 24.0	11.4 ± 24.8	9.5 ± 25.0	1.1 ± 25.1
*p*‐value	–	–	–	–	–	0.128	0.008*	0.031*	0.813
Arm 2: adjuvant CRT	9.0 ± 25.5	15.9 ± 23.3	12.7 ± 24.4	11.7 ± 23.3	12.7 ± 27.2	8.3 ± 19.2	4.8 ± 32.6	3.3 ± 34.4	6.9 ± 32.6
*p*‐value	–	–	–	–	–	0.006*	0.349	0.544	0.228
Overall *p*‐value^a^	0.403	0.470	0.411	0.567	0.384	0.627	0.306	0.372	0.423
EQ‐5D‐3L health state, mean ± SD									
Arm 1: neoadjuvant CRT	73.7 ± 16.5	61.1 ± 19.8	59.5 ± 23.5	70.8 ± 17.7	80.3 ± 17.7	−12.3 ± 20.8	−14.9 ± 25.4	−4.4 ± 16.1	5.0 ± 17.8
*p*‐value	–	–	–	–	–	<0.001*	0.001*	0.118	0.133
Arm 2: adjuvant CRT	72.4 ± 20.5	66.0 ± 14.2	64.5 ± 16.1	68.3 ± 15.8	76.2 ± 16.6	−6.2 ± 19.6	−7.8 ± 19.6	−3.3 ± 21.2	5.1 ± 25.0
*p*‐value	–	–	–	–	–	0.043*	0.013*	0.328	0.239
Overall *p*‐value^a^	0.733	0.209	0.276	0.528	0.338	0.171	0.176	0.798	0.983
EQ‐5D‐3L index (quality‐adjusted‐life‐years), mean ± SD									
Arm 1: neoadjuvant CRT	0.80 ± 0.18	0.70 ± 0.21	0.69 ± 0.24	0.80 ± 0.16	0.87 ± 0.15	−0.10 ± 0.26	−0.11 ± 0.25	−0.02 ± 0.15	0.05 ± 0.20
*p*‐value	–	–	–	–	–	0.021*	0.007*	0.395	0.205
Arm 2: adjuvant CRT	0.84 ± 0.15	0.77 ± 0.15	0.81 ± 0.11	0.82 ± 0.15	0.86 ± 0.19	−0.07 ± 0.18	−0.03 ± 0.16	−0.01 ± 0.15	0.04 ± 0.24
*p*‐value	–	–	–	–	–	0.017*	0.185	0.533	0.312
Overall *p*‐value^a^	0.274	0.102	0.012*	0.695	0.910	0.522	0.095	0.840	0.928

*Note*: *Indicates *p*‐values <0.05 shown as BOLD.

Abbreviations: EORTC, European Organization for Research and Treatment of Cancer; OG25, oesophago‐gastric 25‐item module.

^a^ Reported from two‐sample *t*‐test.

**TABLE 4:** Chemotherapy and Radiation details for all patients and stratified by treatment arm (*n* = 96)

Characteristic	Arm #1: Neoadjuvant CRT (n=47)	Arm #2: Adjuvant CRT (n=49)	p‐value
Chemotherapy^a^ – *n* (%)			
Any	47 (100)	39 (79.6)	**0.001**
Per protocol	24 (51.1)	7 (14.3)	**< 0.001**
Modified	18 (38.3)	20 (40.8)	0.801
Stopped	9 (19.2)	20 (40.8)	**0.021**
Other	1 (2.1)	10 (20.4)	**0.005**
Modified or stopped	23 (48.9)	28 (57.1)	0.421
Modified, stopped or other	23 (48.9)	32 (65.3)	0.105
Chemotherapy completed ≥ 1 cycle – n(%)	47 (100)	33 (67.4)	**< 0.001**
Chemotherapy cycles completed – mean ± SD, median (IQR)	1.9 ± 0.3 2 (2, 2)	3.3 ± 1.0 3.7 (3, 4)	**<0.001** ^**b**^
Cisplatin^a^ – *n* (%)			
Any	47 (100)	35 (71.4)	**<0.001**
Dose reduced	18 (38.3)	12 (24.5)	0.145
Cisplatin infusions – mean ± SD, median (IQR)	7.6 ± 1.1 8 (8, 8)	3.5 ± 1.1 4 (3, 4)	**<0.001** ^**b**^
5‐Fluorouracil^a^ – *n* (%)			
Any	47 (100)	34 (69.4)	**< 0.001**
Dose reduced	17 (36.2)	18 (36.7)	0.954
5‐Fluorouracil infusions – mean ± SD, median (IQR)	2.0 ± 0.5 2 (2, 2)	10.8 ± 1.8 11 (10, 12)	**<0.001** ^**b**^
Epirubicin^a^ – *n* (%)			
Any	0 (0)	34 (69.4)	**<0.001** ^**b**^
Dose reduced	—	9 (18.4)	—
Epirubicin infusions – mean ± SD, median (IQR)	—	2.0 ± 0.8 2 (2, 2)	—
Radiotherapy – n(%)	47 (100)	36 (73.5)	**<0.001**
Radiotherapy completed – n(%)	43 (91.5)	31 (86.1)	0.492
Chemoradiotherapy – n(%)			
Chemoradiotherapy	47 (100)	36 (73.5)	**<0.001**
Chemotherapy only	0 (0)	3 (6.1)	
None	0 (0)	10 (20.4)	
Additional chemotherapy – *n* (%)	1 (2.1)	1 (2.0)	>0.99
Additional radiotherapy – *n* (%)	2 (4.3)	3 (6.1)	>0.99

*Note*: *p*‐values <0.05 shown as BOLD.

Abbreviations: CRT, chemoradiotherapy; IQR, interquartile range.

^a^ Categories not mutually exclusive.

^b^ Anticipated differences due to different protocols.

**TABLE 5:** Surgical details and pathologic outcomes for all patients and stratified by treatment arm (*n* = 96)

Characteristic	*N*	Arm 1: neoadjuvant CRT (*n* = 47)	Arm 2: adjuvant CRT (*n* = 49)	*p*‐value
Surgery, *n* (%)	96	41 (87.2)	49 (100)	0.012*
Neck incision, *n* (%)				
Left	90	32 (78.1)	40 (81.6)	0.672
None		9 (22.0)	9 (18.4)	
Chest incision, *n* (%)				
VATS		5 (12.2)	8 (16.3)	0.556
Thoracotomy		5 (12.2)	2 (4.1)	
VATS to thoracotomy	90	2 (4.9)	3 (6.1)	
None		29 (70.7)	36 (73.5)	
Abdomen incision, *n* (%)				
Laparoscopic	90	37 (90.2)	42 (85.7)	0.824
Laparotomy		3 (7.3)	3 (6.1)	
Laparoscopic to laparotomy		1 (2.4)	3 (6.1)	
None		0 (0)	1 (2.0)	
Primary surgery, *n* (%)				
Esophagectomy‐gastric interposition	90	38 (92.7)	44 (89.8)	0.723
Exploration		3 (7.3)	5 (10.2)	
Anastomosis, *n* (%)				
Neck	90	32 (78.1)	40 (81.6)	0.731
Chest		6 (14.6)	4 (8.2)	
None		3 (7.3)	5 (10.2)	
Unresectable, *n* (%)	90	2 (4.9)	4 (8.2)	0.685
Pathological stage, *n* (%)				
T0N0	86	8 (21.1)	0 (0)	–
T1N0		7 (18.4)	5 (10.4)	
T1N1		2 (5.3)	3 (6.3)	
T2N0		5 (13.2)	1 (2.1)	
T2N1		1 (2.6)	1 (2.1)	
T3N0		11 (29.0)	9 (18.8)	
T3N1		4 (10.5)	10 (20.8)	
T3N2		0 (0)	7 (14.6)	
T3N3		0 (0)	11 (22.9)	
T4N1		0 (0)	1 (2.1)	
Tumor grade, *n* (%)				
I	73	3 (10.7)	6 (13.3)	0.909
II		15 (53.6)	22 (48.9)	
III		10 (35.7)	17 (37.8)	
Positive margins,^a^ *n* (%)				
Any	83	2 (5.3)	20 (44.4)	<0.001*
Proximal		1 (2.6)	0 (0)	0.458
Distal		0 (0)	1 (2.2)	>0.99
Radial		1 (2.6)	20 (44.4)	<0.001*
Resection status, *n* (%)				
R0 (“complete”)	90	37 (90.2)	25 (51.0)	<0.001*
R1 (“microscopic/margin positive”)		2 (4.9)	20 (40.8)	
R2 (“unresectable”)		2 (4.9)	4 (8.2)	
Positive nodes (%), mean ± SD,	83	4.8 ± 12.8	28.8 ± 30.2	<0.001*
Median (IQR)		0.0 (0.0, 0.0)	22.2 (0.0, 40.0)	
Invasion^a^, *n* (%)				
Any	83	15 (39.5)	37 (82.2)	<0.001*
Stomach		10 (26.3)	20 (44.4)	0.087
Lymphovascular		5 (13.2)	31 (68.9)	<0.001*
Venous		1 (2.6)	13 (28.9)	0.002*
Gastric/serosal		0 (0)	1 (2.2)	>0.99
Perineural		2 (5.3)	14 (31.1)	0.003*
Feeding tube inserted^a^, *n* (%)				
Any		43 (91.5)	47 (95.9)	0.431
Chemoradiotherapy		23 (48.9)	5 (10.2)	<0.001*
Before surgery	96	28 (59.6)	0 (0)	<0.001*
Post‐operative stay		14 (29.8)	8 (16.3)	0.117
1‐month post‐discharge		15 (31.9)	5 (10.2)	0.009*
Deceased, *n* (%)	96	31 (66.0)	38 (77.6)	0.207
90‐day mortality, *n* (%)	96	1 (2.1)	5 (10.2)	0.204
Status, *n* (%)				
Alive without disease	96	14 (29.8)	10 (20.4)	0.461
Alive with disease		2 (4.3)	1 (2.0)	
Deceased		31 (66.0)	38 (77.6)	
Recurrence, *n* (%)	96	29 (61.7)	27 (55.1)	0.512
Local recurrence, *n* (%)	96	6 (12.8)	5 (10.2)	0.694
Regional recurrence, *n* (%)	96	3 (6.4)	4 (8.2)	>0.99
Distant recurrence, *n* (%)	96	24 (51.1)	22 (44.9)	0.546
Recurrence pattern, *n* (%)				
Local	56	5 (17.2)	3 (11.1)	–
Regional		0 (0)	1 (3.7)	
Distant		20 (69.0)	19 (70.4)	
Local + regional		0 (0)	1 (3.7)	
Local + distant		1 (3.5)	1 (3.7)	
Regional + distant		3 (10.3)	2 (7.4)	
Adverse events^a^, *n* (%)				
Chemoradiotherapy + grade ≥2	96	47 (100)	34 (69.4)	<0.001*
Chemoradiotherapy + grade ≥3		37 (78.7)	27 (55.1)	0.014*
Surgery + grade ≥2		34 (72.3)	42 (85.7)	0.107
Surgery + grade ≥3		27 (57.5)	37 (75.5)	0.061

*Note*: *Indicates *p*‐values <0.05.

Abbreviations: CRT, chemoradiotherapy; IQR, interquartile range; VATS, video‐assisted thoracoscopic surgery.

^a^ Categories not mutually exclusive.

### Pathological findings

Adenocarcinomas were the most common (89% vs. 90%) followed by squamous cell (4% vs. 10%) with unknown histology for 3 (6%) patients in the neoadjuvant arm. A total of 92% of patients in the neoadjuvant arm had a complete R0 resection compared to only 51% in the adjuvant arm (*p* < 0.001). A pathological complete response (ypT0N0) was observed in the resection specimens from 8 (21%) patients in the neoadjuvant arm all corresponding with adenocarcinomas. Regional lymph nodes were positive in 5% of neoadjuvant patients compared to 29% in the adjuvant arm (*p* < 0.001) (Table 5).

### Survival

**FIGURE 5:**
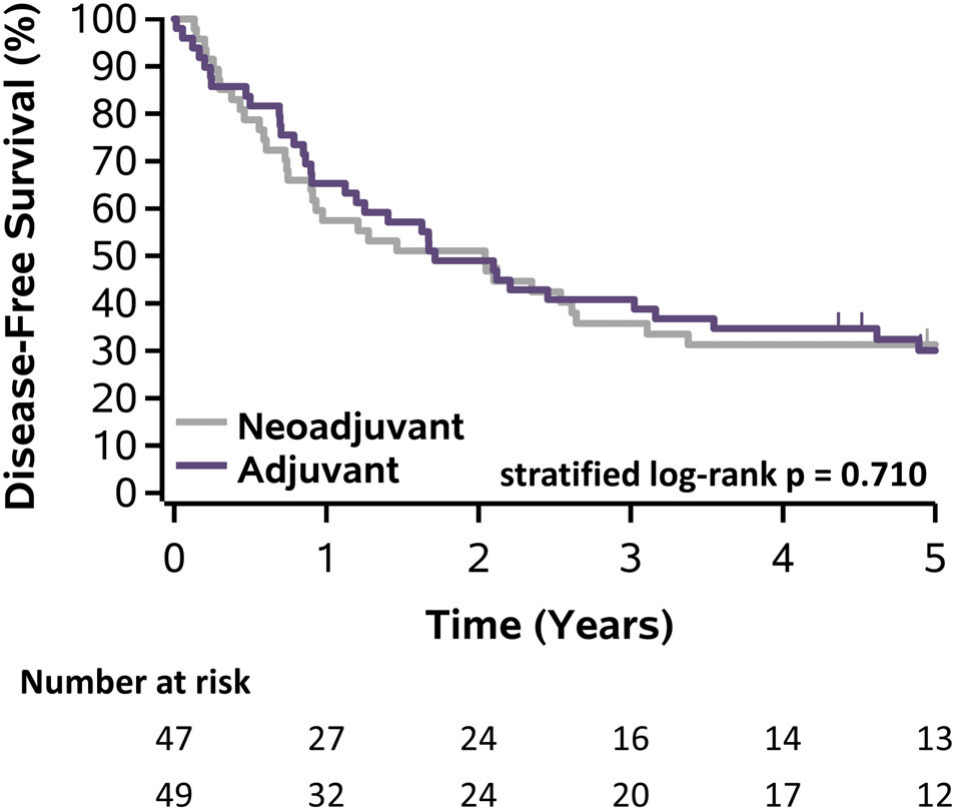
Kaplan‐Meier plot of disease‐free survival stratified by treatment arm

There were no 30‐day mortalities in either arm, but there was 1 (2%) 90‐day mortality in the neoadjuvant arm and 5 (10%) 90‐day mortalities in the adjuvant arm (*p* = 0.204), which is not surprising because the trial was powered to detect a difference. There were no significant differences comparing neoadjuvant and adjuvant arms in either OS (*p* = 0.409), 5‐year OS 35% vs. 32%, or DFS (*p* = 0.710), 5‐year DFS 31% vs. 30% (Figures 4 and 5). Despite extended field radiotherapy, there were no significant differences in local (13% vs. 10%, *p* = 0.694), regional (6% vs. 8%, *p* > 0.99), or distant recurrences (51% vs. 45%, *p* = 0.546) between the neoadjuvant and adjuvant arms, respectively (Table 5).

### Adverse events

There was also significant morbidity for all patients undergoing trimodality treatment. Every patient experienced at least one adverse event. Clinically significant adverse events (grade ≥2) attributed to chemoradiotherapy occurred in 100% of neoadjuvant and in 69% of adjuvant patients (*p* < 0.001), whereas serious adverse events (grade ≥3) occurred in 79% of neoadjuvant and 55% of adjuvant patients (*p* = 0.014), more frequently in neoadjuvant arm (Table 5). Abdominal pain, dehydration, esophagitis, and febrile neutropenia related to chemoradiotherapy were significantly more frequent in the neoadjuvant arm (*p* < 0.05), whereas hypokalemia and hypomagnesemia related to chemoradiotherapy were significantly more frequent in the adjuvant arm (*p* < 0.05) (Table S2 in Appendix). When a per protocol analysis was performed using only the 36 patients that received any chemoradiation in the adjuvant arm then 94% of patients endured a clinically significant adverse event (grade ≥2), and there was no difference between the arms. In contrast, clinically significant adverse events attributed to surgery occurred in 72% of neoadjuvant and 86% of adjuvant patients (*p* = 0.107) and serious adverse events in 58% of neoadjuvant and 76% of adjuvant (*p* = 0.061), more frequently in adjuvant arm. Catheter infections and esophageal stenosis related to surgery were more frequent in the adjuvant arm (*p* < 0.05), whereas gastroesophageal reflux and pneumonia were more frequent in the neoadjuvant arm (*p* < 0.05). More specifically, esophageal anastomotic leak grade ≥2 was similar between the neoadjuvant arm (13%, *n* = 6) and the adjuvant arm (16%, *n* = 8) (*p* = 0.947); however, esophageal stenosis grade ≥2 was more frequent in the adjuvant arm (41%, *n* = 20) compared to the neoadjuvant arm (15%, *n* = 7) (*p* = 0.007) (Table S3 in Appendix).

## DISCUSSION

We assessed the HRQOL of patients with esophageal cancer undergoing two different trimodality protocols over 1 year and found no clinical or statistically significant differences between the neoadjuvant and adjuvant protocols. At 1‐year follow‐up the mean FACT‐E scores improved significantly compared to baseline, but they did not reach the a priori minimal clinically important difference threshold of 15 points.

During the first year of trimodality treatment there was, however, a clinically and statistically significant greater decrease in the FACT‐E scores in the neoadjuvant arm at 2 months compared to the adjuvant arm with similar trends observed for FACT‐G, EORTC QLQ‐OG25, and EQ‐5D‐3L instruments that surpassed their MCID. Most patients completed their chemoradiation before 4 months and all were done before 6 months. The declines were mostly in the dysphagia, reflux, pain, taste, and coughing domains. We hypothesize this may be because of the delay in the relief of symptoms because of the inflammatory effects of radiation on the esophagus before surgery. All patients in the neoadjuvant arm received some radiation, of which 92% completed the prescribed dose compared to only 74% receiving some radiation in the adjuvant arm. It is well known that radiotherapy can cause numerous complications, including esophagitis, pneumonitis, and anorexia.^30^, ^31^ We speculate that patients in the adjuvant arm that underwent surgery first had their swallowing restored by 2 months and therefore, reported less dysphagia. At 1 year, most patients had recovered from the toxicities of trimodality treatment and their HRQOL became better overall statistically, but below the MCID of 15 threshold. Our results appear to indicate that neoadjuvant therapy produced a significant decrease in HRQOL for several months during the initial phase of their trimodality treatment.

Our study is the only randomized trial that used the disease‐specific outcomes captured in the FACT‐E instrument to assess HRQOL in esophageal cancer patients undergoing trimodality therapy. Scarpa et al.^2^ systematically reviewed the HRQOL after esophagectomy for cancer that included 21 observational studies comprising a total of 1282 patients.^2^ The analysis using EORTC QLQ‐C30 during a 6‐month follow‐up showed that the global scale and physical function decreased from baseline. van den Boorn et al.^4^ performed a systematic review of 49 studies to evaluate the HRQOL in curatively‐treated patients with esophageal or gastric cancer, but the majority (61%) of the studies unfortunately were of low quality.^4^ Their meta‐analysis of 36 of the studies that used the EORTC questionnaires showed similar short‐term HRQOL changes in esophageal cancer patients receiving definitive chemoradiotherapy, neoadjuvant chemoradiotherapy/chemotherapy, or surgery alone. The combined overall neoadjuvant experiences were like our own, however, they found no studies that evaluated the effects of adjuvant chemoradiation. In fact, there is only one other randomized trial that has investigated the role of neoadjuvant compared to adjuvant trimodality therapy in the treatment of locally advanced carcinoma of the esophagus, but unfortunately it included only squamous cell carcinomas and it did not report on any HRQOL outcomes.^5^

To our knowledge, there has been no randomized trial evaluating neoadjuvant versus adjuvant chemoradiation for adenocarcinomas of the esophagus and gastroesophageal junction. Our trial evaluated two different perioperative protocols. The neoadjuvant arm consisted of cisplatin and 5‐FU plus radiation, which was the current standard at the time. Our adjuvant arm consisted of ECF chemotherapy plus radiation, which was adopted from our gastric cancer protocol at the time.^7^, ^14^, ^32^ In our trial, chemoradiotherapy related adverse events were significantly more frequent in the neoadjuvant arm. There is substantial evidence in the literature to suggest that neoadjuvant chemoradiation increases the rate of postoperative morbidity and mortality. A meta‐analysis of randomized trials of patients with resectable esophageal cancer showed that neoadjuvant chemoradiation was a risk factor for postoperative mortality.^30^, ^33^, ^34^, ^35^ It was reassuring that we found similar anastomotic leaks in the neoadjuvant arm compared to the adjuvant arm, but we did find significantly more strictures in adjuvant arm. Our own previous experience showed that extended field radiation decreased local recurrence rates; however, our current trial found no differences in local, regional, or even distant recurrence rates between the two arms.^36^

We observed no significant differences in OS and DFS between arms with comparable 5‐year rates. Most of the evidence currently suggests the optimal treatment for resectable esophageal cancer should consist of trimodality therapy, but the sequencing remains unclear.^38^, ^39^, ^40^ adjuvant therapies appear to be less effective compared with the neoadjuvant approaches and do not lead to an improvement in OS when compared with surgery alone.^39^ In contrast to data available on neoadjuvant chemoradiotherapy, there are only two randomized studies comparing adjuvant chemotherapy plus radiotherapy with surgery alone for esophageal cancer.^5^, ^40^ MacDonald et al.^40^ compared surgery alone with surgery and adjuvant 5‐FU chemotherapy plus 45 Gy radiation for patients with resectable adenocarcinomas of the stomach and gastroesophageal junction, of which only 20% of the patients had cancers of the gastroesophageal junction. The median OS in the surgery only group was 27 months, as compared with 36 months in the chemoradiotherapy group (*p* = 0.005). Our 35% 5‐year OS in the neoadjuvant arm compares favorably with the 36% from the Medical Research Council Adjuvant Gastric Infusional Chemotherapy (MAGIC) trial, whereas our 32% 5‐year OS in the adjuvant arm is inferior to the 42% from MacDonald et al.^40^ that included predominately gastric cancers.^41^

Lv et al.^5^ investigated the role of neoadjuvant and adjuvant trimodality therapy in the treatment of locally advanced squamous cell carcinoma of the esophagus by randomizing patients into 3 groups: preoperative chemoradiation (*n* = 80), postoperative chemoradiation (*n* = 78), and surgery alone (*n* = 80) and found no significant differences in OS and progression‐free survival, but there were fewer complications with postoperative chemoradiation. Our neoadjuvant arm OS rates were slightly inferior to the 44% 5‐year OS reported by Lv et al.^5^, but this trial used the more modern paclitaxel and cisplatin chemotherapy and only included squamous cell carcinomas of the esophagus. Van Hagan et al.^42^ found a 47% 5‐year OS for neoadjuvant treatment comprised of carboplatin, paclitaxel, and radiation (chemoradiotherapy for esophageal cancer followed by surgery study [CROSS] protocol) in predominately adenocarcinomas of the esophagus. This combination has become the current standard among most oncologists for adenocarcinomas of the esophagus and gastroesophageal junction. The role of radiation is currently being assessed in several randomized trials and may prove not be necessary.^43^, ^44^, ^45^

There are several limitations of our study that should be noted. Our trial is based on a relatively small sample size with a reduced power to detect statistical differences in OS and DFS, but we did not expect there would be a difference. We hypothesized that both trimodality treatment options would provide similar survival results, but have different patient‐centered experiences with respect to HRQOL and adverse events. Our trial was adequately powered to detect a difference in the FACT‐E if difference exists. It turned out there was no difference overall, but there were differences in HRQOL and adverse events. The HRQOL was significantly inferior at 2 months in the neoadjuvant arm for FACT‐E, EORTC QLQ‐OG25, and EuroQol 5‐D‐3 L in the dysphagia, reflux, pain, taste, and coughing domains. Half of the patients were able to complete the prescribed neoadjuvant arm chemotherapy without modification compared to only 14% in the adjuvant arm. Chemotherapy related adverse events occurred significantly more frequently in the neoadjuvant arm.

Our study was a pragmatic trial of our ECF adjuvant trimodality protocol compared to the more common cisplatin and 5‐FU neoadjuvant protocol. It was not designed to compare the timing of similar chemoradiation protocols. The cisplatin dose for the neoadjuvant arm was 100 mg/m^2^ per cycle, a dose also used in the CALGB 9781 study.^46^ However, most centers and guidelines now propose 75 mg/m^2^ of cisplatin per cycle so the toxicities seen in the neoadjuvant arm from chemotherapy are likely more severe than those observed in current clinical practice, which has evolved since CALGB 9781 was published. We acknowledge that our two chemotherapy protocols are now considered obsolete in many centers in North America and Europe, but they were the standard of care at the inception of the trial and continue to be used elsewhere in the world. We recognize that some patients with stage I cancers were included in the neoadjuvant chemoradiation arm and may have been overtreated. This could have biased the results in favor for this arm, but this was not the case. We acknowledge the limitations of clinical staging, but felt it was appropriate to not deny patients the benefit of induction therapy if they were clinically under staged.

The methodological strengths of our trial include the prospective randomized design and complete follow‐up data for all participants. The HRQOL data were based on multiple validated instruments that covered the spectrum of HRQOL research from disease‐specific to utility measurements. The adverse event data were collected every 2 months for 1 year following randomization that allowed for a detailed longitudinal investigation of the changes in HRQOL over time between arms. This is the only randomized trial evaluating neoadjuvant versus adjuvant chemoradiation for predominately adenocarcinomas of the esophagus and gastroesophageal junction that used HRQOL as the primary outcome measure.

## CONCLUSIONS

Neoadjuvant cisplatin and 5‐FU chemotherapy with radiation followed by surgery and adjuvant epirubicin, 5‐FU, and cisplatin chemotherapy with extended field radiation therapy are both challenging for patients, but provide similar survival benefits with different HRQOL experiences in patients with resectable esophageal cancer. More effective newer combinations and scheduling of systemic therapy, radiation, and surgery should be explored to improve survival and health related quality‐of‐life while and minimizing adverse effects.

## CONFLICT OF INTEREST

The authors do not have any conflict of interest to disclose with respect to this study.

## ETHICS APPROVAL

The study was completed and approved by our local ethics review board in accordance with the declaration of Helsinki, Good Clinical Practice guideline.

## PATIENT CONSENT

All included patients provided written informed consent.

## REGISTRATION

ClinicalTrials.gov (NCT00907543).

## Supporting information


**Appendix** **S1**

Table S1

Table S2

Table S3
