# Supplementary material for: The quality of life in neoadjuvant versus adjuvant therapy of esophageal cancer treatment trial (QUINTETT): Randomized parallel clinical superiority trial
Source: Thorac Cancer. 2022 May 24;13(13):1898–915. doi: 10.1111/1759-7714.14433 (PMC9250846; doi:10.1111/1759-7714.14433)
Supplement: Supplementary file 1 — Appendix S1 [file TCA-13-1898-s001.docx]

**A Prospective Randomized Phase III Trial Comparing**

**Preoperative Chemoradiation Therapy**

**(Cisplatin, 5-FU and Radiotherapy followed by Surgery)**

**to Surgery followed by Postoperative Chemoradiation**

**(Cisplatin, Epirubicin, 5-FU, Radiotherapy)**

**for Esophageal Cancer**

**Thoracic Multi-Discipline Team**

**London Regional Cancer Program**

Principal Investigator:

Richard A. Malthaner MD MSc FRCSC FACS FCCP

Associate Professor of Surgery

Director of Thoracic Surgery Research

The University of Western Ontario

London Health Sciences Centre

800 Commissioners Road East, Room E2-124

London, Ontario

N6A 5W9

Telephone: (519) 667-6835

Fax: (519) 667-6517

Email: richard.malthaner@lhsc.on.ca

1.0 Introduction 4

2.0 Hypothesis 7

3.0 Objectives 7

3.1 Primary 7

3.2 Secondary 7

4.0 Primary Endpoint 7

4.1 FACT-E 8

4.2 EORTC QLQ-OG25 8

4.3EQ-5D 9

5.0 Research Design & Methods 9

5.1 Eligibility 9

6.0 Screening & Randomization 11

6.1 Recruitment 11

6.2 Initial Screening 11

6.3 Eligibility visit pre-randomization 11

7.0 Randomization 12

8.0 Surgical Treatment 12

8.1Standardization of technique 12

8.2 Surgical Technique 12

8.3 Surgical Quality assurance 14

9.0 Chemotherapy 14

9.1 Cisplatin 15

9.2 5FU 16

9.3 Epirubicin 18

9.4 Dose Modification & Management of Toxicity 18

10.0 Radiation 21

11.0 Outcomes 25

12.0 Follow-up & Data Collection 27

13.0 Central Adjudication of Events 27

14.0 Adverse Events & Safety 27

15.0 Management of Intercurrent Events 27

16.0 Participant Safety/Confidentiality 28

17.0 Sample Size 28

18.0 Trial Organization 30

19.0 Publications 30

20.0 Feasibility of Recruitment 30

21.0 Limitations/Pitfalls 30

Study Schema 32

Appendices 33

References 34

RESEARCH PROPOSAL:

**1.0 Introduction**

Carcinoma of the esophagus is an aggressive malignancy that continues to kill more than 90% of people with the disease within five years (1). The incidence of adenocarcinoma of the esophagus is rising faster than any other malignancy (2). Its virulence, in terms of symptoms and mortality, justifies a continued search for optimal therapy.

Any treatment modality chosen for esophageal cancer appears to depend on local practices. The available therapeutic options are surgery, chemotherapy, radiotherapy, and various combinations of these modalities. Recent advances in the multi-modality approach have brought about improved outcomes, but five-year survival rates remain less than 20% (3). Reasons for this poor survival include the advanced state of the disease before symptoms occur, frequent lymph node involvement (occurring in up to 60% of presenting patients), and the common occurrence of submucosal spread and extension to surrounding structures. In addition, there is no satisfactory routinely accepted method of preoperatively staging esophageal cancer.

The most recent practice guideline from Cancer Care Ontario states: “Based on the majority of the evidence available at this time, the Gastrointestinal Cancer Disease Site Group believes that preoperative chemoradiotherapy for resectable carcinoma of the esophagus is the preferred approach (4). The key evidence is drawn from a literature meta-analysis of 10 randomized trials comparing preoperative chemoradiotherapy followed by surgery to surgery alone showed a 13% absolute benefit in survival at 2 years for preoperative chemoradiotherapy (HR= 0.81; 95% CI 0.70-0.93; p=0.002) (5). A published abstract of an individual patient data (IPD)-based meta-analysis of 9 randomized trials (2,102 patients) comparing preoperative chemotherapy followed by surgery to surgery alone demonstrated a 4% (from 16 to 20%) absolute overall survival advantage for chemotherapy at 5-years (HR-0.87, 95% CI 0.79-0.95, p=0.003). Based on 7 trials (1,849 patients), the HR for disease free survival (DFS) was 0.82 (95% CI 0.74-0.91, p=0.001) in favour of CT+S, representing a 5-year absolute DFS benefit of 4% (from 6 to 10%). No difference was seen in postoperative death (6.7%) (6). Randomized trials demonstrated no survival benefit for radiotherapy given alone, either preoperatively or postoperatively, compared with surgery alone. Randomized trials demonstrated no survival benefit for postoperative chemotherapy given alone compared with surgery alone. The new standard of care for esophageal cancer appears to be preoperative chemoradiation followed by surgery for patients with operable and resectable esophageal cancer (4).

No randomized trial has evaluated postoperative chemotherapy combined with radiation versus surgery alone for esophageal cancer. Recently, the intergroup study INT-0116 randomized 556 patients, following curative resection of gastric cancer, to either postoperative combined chemoradiotherapy or observation alone (7). The treatment consisted of one cycle of 5-FU (425 mg/m2/day) and leucovorin (20 mg/m2/day) in a daily x 5 regimen followed one month later by 45 Gy (1.8 Gy/day) of radiation given with 5-FU (400 mg/m2/day) and leucovorin (20 mg/m2/day) on days 1 through 4 and the last 3 days of radiation. One month after completion of radiation, two cycles of daily x5 5-FU (425 mg/m2/day and leucovorin (20 mg/m2/day) were given at monthly intervals. Most tumours were in the distal stomach; however, tumours were present in the gastroesophageal junction in approximately 20% of patients. More than two thirds of them had either T3 or T4 tumours, and 85% had nodal metastases. The median overall survival in the surgery only group was 27 months, as compared with 36 months in the chemoradiotherapy group. The hazard ratio for death was 1.35 (95% confidence interval, 1.09 to 1.66; p=0.005). Three patients (1%) died from toxic effects of the chemoradiotherapy, and grade 3 toxic effects occurred in 41% and grade toxic effects occurred in 32%. There was a 12% survival advantage at five years with combined modality treatment reached 40%. Cancer Care Ontario now recommends postoperative chemoradiation for T3-4, N1 resected gastric cancers (8).

There have been several case series that examined the role of postoperative chemoradiation for esophageal cancer (9–12). Saito and colleagues reported on 35 patients that underwent transthoracic esophagectomy with lymphadenectomy and were subsequently treated with 50 Gy or radiation and two courses of concurrent chemotherapy (cisplatin, vindesine and pepleomycin) (9). The tolerance in all patients was good and the five-year survival rate was 31%.

Our own phase II data suggested that postoperative chemoradiation may be beneficial for esophageal cancer patients (10). Thirty-eight lymph node positive (N1) patients underwent esophagectomy followed by postoperative chemotherapy (cisplatin, 5-FU, + epirubicin) and 50 Gy radiotherapy (CRT) and 28 (N1 patients) underwent surgery alone (S). Pre-operative risk factors, tumour characteristics, ECOG scores and lengths of stay were similar. Disease-free survival was similar (CRT 10.2 months vs. S 10.6 months) but the surgery alone group had more local recurrences (CRT 13% vs. S 35%). Overall Kaplan-Meier survival analysis showed a significant advantage with postoperative chemoradiation (log rank p=0.001). The median overall survival for the chemoradiation group was 47.5 months which was significantly longer than the surgery alone group (14.1 months). The three-year survival was 62% in the CRT group and 25% in the S group, and the five-year survival was 48% and 0% respectively.

Rice and colleagues from the Cleveland Clinic compared 31 patients with locoregionally advanced esophageal cancer (90% pT3, 81% pN1, and 13% pM1a) that received postoperative adjuvant chemoradiotherapy after resection to 52 patients that underwent esophagectomy alone (11). Patients received 50.4 to 59.4 Gy external beam radiation with concurrent two 4-day cycles of intravenous 5-fluorouracil (1,000 mg/m2/d) and cisplatin (20 mg/m2/d) during the first and fourth week of adjuvant therapy. Among propensity-matched patients, median, 1-year, and 4-year survivals for those receiving adjuvant therapy versus esophagectomy were 28 versus 15 months, 60% + 11% versus 65% + 11%, and 44%+11% versus 0% (P=0.05). Median time to recurrence was 25 versus 13 months (P =0.04), and recurrence-free survival was 22 versus 10 months (P=0.02). The authors concluded that patients with locoregionally advanced carcinoma after esophagectomy should be considered for adjuvant therapy.

In a more recent cohort series report, Liu et al from China compared patients with T3-4 and N0-1 esophageal cancer that underwent either post-operative concurrent chemoradiotherapy with weekly cisplatin 30 mg/m2 followed by systemic adjuvant chemotherapy (four monthly cycles of cisplatin 20 mg/m2 and 5-fluorouracil 1 000 mg/m2 for five consecutive days), or, post-operative radiation alone (12). The radiotherapy dose was 55-60 Gy for all patients. A total of 60 patients (n = 30 per group) were enrolled in this study. The two groups were generally comparable for demographic characteristics and hematological and non-hematological toxicities. The treatment with weekly cisplatin was well tolerated, with significantly better mean overall survival (30.9 months vs 20.7 months; 95% CI, 27.5-36.4 vs 15.2-26.1) and 3-year survival (70.0% vs 33.7%; P = 0.003). Low histological grade of tumor (P<0.001) was associated with favorable survival in these locally advanced patients. They concluded for locally advanced esophageal cancer, the combination of esophagectomy, post-operative chemoradiotherapy with weekly cisplatin and systemic adjuvant chemotherapy was well tolerated and effective.

Optimal radiation therapy target volume definition for esophageal cancer patients receiving primary chemoradiation therapy is controversial, partly due to significant changes in patient anatomy and function, and partly due to limited information available to evaluate the benefit on anastomotic site coverage in the post-surgical radiation therapy target volume. Our own experience (13) showed a significant reduction in local recurrence using extended volume external beam radiation therapy covering the anastomotic site in post – esophagectomy high risk patients.

There has never been a direct comparison of preoperative chemoradiation to postoperative chemoradiation for esophageal cancer. We propose to determine if there is an advantage for either preoperative chemotherapy and radiotherapy followed by surgery or surgery followed by postoperative chemotherapy and radiotherapy. The study builds upon the results of the previous intergroup trials, three phase II trials and our own experience and represents the next logical treatment option.

**2.0 Hypothesis**

Postoperative cisplatin, 5-FU, and epirubicin chemotherapy with concurrent extended volume radiotherapy following surgical resection is superior to standard preoperative cisplatin and 5-FU chemotherapy plus radiotherapy followed by surgical resection for resectable esophageal carcinoma.

**3.0 Objectives**

The main objective is to compare adjuvant cisplatin, epirubicin, and 5-FU with concurrent extended volume radiotherapy following surgical resection (adjuvant trimodality therapy) to preoperative cisplatin and 5-FU chemotherapy and surgery (induction trimodality therapy).

**3.1 Primary Objectives**

3.1.1 To compare the disease specific health-related quality-of-life using the FACT-E between the two treatments.

**3.2 Secondary Objectives**

3.2.1 To assess and compare the toxicities and morbidities of each approach.

3.2.2 To compare overall survival rates between the two treatment arms.

3.2.3 To compare recurrence in the two groups including local (gastric or esophageal bed), regional (regional lymph nodes), and distant recurrences (supraclavicular lymph node, liver, peritoneal carcinomatosis, or lung, brain, etc.).

3.2.4 To conduct an economic analysis comparing the two treatments.

3.2.5 To compare the proportion of complete (R0), microscopic positive (R1), and grossly positive (R2) resections between treatment arms.

**4.0 Primary Endpoint:**

**Health-Related Quality-of-Life**

Three health-related quality-of-life instruments will be used. They will include a utility instrument, a generic health index, and two disease-specific instruments, representing the full spectrum of different approaches to Health-Related Quality of Life (HRQOL) measurement. The primary outcome will be the FACT-E.

**4.1 The Functional Assessment of Cancer Therapy – Esophageal (FACT-E)**

The Functional Assessment of Cancer Therapy-General (FACT-G) is a self-administered 27 item general cancer quality of life (QL) measure for evaluating patients receiving cancer treatment (14,15). The FACT G meets or exceeds all requirements for use in oncology clinical trials, including ease of administration, brevity, reliability, validity, and responsiveness to clinical change (16). It is divided into four primary domains: physical well-being, social / family wellbeing, emotional wellbeing, and functional well-being.

The FACT-E has recently been developed and validated (17). It is a quality-of-life subscale for patients with esophageal cancer to be used with the FACT-G and provides a more multidimensional assessment of HRQOL than the EORTC QLQ-C30/QLQ-OG25 (18–22). It captures the clinically relevant problems associated with esophageal cancer patients and takes less than 10 minutes to administer. The FACT-G consists of 27 items divided among 4 subscales: physical well-being (PWB), functional well-being (FWB), social/family well-being (SWB), and emotional well-being (EWB). The sum of these subscales forms the total FACT-G score. The FACT-G has been well studied and previously validated (19). The Esophageal Cancer Subscale (ECS) of the FACT-E addresses concerns specific to patients with esophageal cancer such as eating, appetite, swallowing, pain, talking/communicating, mouth dryness, breathing difficulty, coughing, and weight loss. The total FACT-E score is the sum of the ECS and the FACT-G scores. A trial outcome index (TOI) score, useful where the physical domains are of interest, is computed by adding the PWB and FWB scores to the ECS score. All FACT-E items are rated on a 5-point Likert scale ranging from 0 = ‘‘not at all’’ to 4 = ‘‘very much’’. Negatively worded items are reverse scored so that higher scores always represent better quality of life (QOL) or less severe symptoms. Possible score ranges are PWB (0–28), FWB (0–28), SWB (0–28), EWB (0–24), ECS (0–68), TOI (0–124), swallowing index (0–20), eating index (0–12), FACT-G (0–108), and FACT-E (0–176).Yost and Eton have reviewed combining distribution- and anchor-based approaches to determine minimal clinically important difference values for the various FACT instruments by disease site and suggested a range between 5% and 10%, translating to a range of 8.8 to 17.6 points for FACT-E based on a total score of 176 points (23). The a priori minimal clinically important difference was conservatively set at 15 indicating that a 15-point increase in the FACT-E score compared to baseline would correspond with a clinically meaningful positive effect on HRQOL for the patient (24).

A prospective cohort study of patients with esophageal cancer treated with surgery alone or neoadjuvant chemoradiotherapy and surgery evaluating the validity, internal consistency, and responsiveness to change of the FACT-Esophageal (FACT-E) when comparing it with the European Organization for the Research and Treatment of Cancer Quality of Life Questionnaire (EORTC QLQ 30) and esophageal (OES 24) as well as clinical factors. The FACT-E demonstrated very good convergent and divergent validity when compared with the EORTC QLQ30 and OES 24 and clinical variables. Internal consistency was also good with coefficient alpha > 0.70 for all subscales and individual items. Stability coefficients were > 0.80. Changes in clinical status were reflected in changes in FACT-E scores demonstrating responsiveness to change, particularly in patients receiving neoadjuvant chemoradiotherapy before surgery. The FACT-E met or exceeded all standards for validity, providing an option to measure health-related quality of life for different treatment strategies for esophageal cancer. The FACT-E contains a summative domain with standard deviation and clinical effect size information that can be used for sample size calculation.

**4.2 The EORTC QLQ-OG25**

The most recent instrument is the EORTC QLQ-OG (18). It combines the EORTC questionnaires for assessing quality of life (HRQL) for esophageal (QLQ-OES18) and stomach cancer (QLQ-STO22), into a single questionnaire for tumours of the esophagus, Oesophago-gastric junction, or stomach. The QLQ-OES18, QLQ-STO22 and seven modified items were administered to 300 patients with oesophageal (n=148), junctional (n=66), or gastric cancer (n=86). Semi-structured interviews assessed item and scale preference and multi-trait scaling analyses confirmed the scale structure of the new module (QLQ-OG25). This was further tested for validity. The QLQ-OG25 has six scales, dysphagia, eating restrictions, reflux, odynophagia, pain, and anxiety. Scales have good reliability (alpha range 0.67-0.87), and they distinguish between tumour sites and disease stage. Scales do not correlate highly with scores from the core questionnaire, thus indicating that the module was addressing separate HRQL aspects. Questionnaire responses were transformed linearly into scores ranging from 0 to100 according to the EORTC scoring manual (28). A higher score indicates either more symptoms or better function, depending on the question. The minimal clinically important difference is estimated to be 10 (24). The QLQ-OG25 is recommended to supplement the EORTC QLQ-C30 when assessing HRQL in patients with esophageal, junctional, or gastric cancer. The FACT-E and EORTC QLQ-OG25 questionnaires assess similar generic aspects of quality of life. The correlation between them is poor except for the swallowing domain (19). We therefore believe that both instruments should be used.

**4.3 EuroQoL EQ-5D**

EQ-5D is a standardized instrument for use as a measure of health outcome (20). It is applicable to a wide range of health conditions and treatments; it provides a simple descriptive profile and a single index value for health status. EQ-5D was originally designed to complement other instruments but is now increasingly used as a 'standalone' measure (21). EQ-5D is designed for self-completion and is cognitively simple, taking only a few minutes to complete. Instructions to respondents are included in the questionnaire. The current 3-level, 5-dimensional format of the EQ-5D will remain unchanged for the immediate future. EQ-5D is one of a handful of measures recommended for use in cost-effectiveness analyses by the Washington Panel on Cost Effectiveness in Health & Medicine (22–24). It has also been used in cancer patients and the minimally important differences has been estimated to be 0.06 for US-index scores (25).

**5.0 Research Design and Methods**

**5.1 Eligibility Criteria**

5.1.1 Histologically documented squamous cell carcinoma or adenocarcinoma of the thoracic esophagus (> 20 cm from the incisors) or gastroesophageal junction are included. Cancers of the cervical esophagus (< 20 cm are excluded).

5.1.2 No distant metastases (M0).

5.1.3 Patients will be stratified by stage (clinical N0 versus clinical N1), and surgeon (RI versus RM versus DF)

5.1.4 Patients with tumours within 3 cm distal spread into gastric cardia as detected by esophagogastroscopy. Tumours that have > 3 cm of spread into cardia of the stomach are considered gastric cancers and are ineligible.

5.1.5 Patients with biopsy (by endoscopic ultrasound, laparoscopy, or laparotomy) proven metastatic supraclavicular nodes are ineligible.

5.1.6 Patients with biopsy proven metastatic celiac nodes are ineligible. Resectable mediastinal nodes are eligible.

5.1.7 No prior chemotherapy for this malignancy.

5.1.8 No prior radiotherapy that would overlap the field(s) treated in this study.

5.1.9 Patients with other malignancies are eligible only if > 5 years without evidence of disease or completely resected or treated non-melanoma skin cancer.

5.1.10 Age > 18 years and able to tolerate tri-modality therapy at the discretion of the treating thoracic surgeon, medical and radiation oncologists. Tumours must be resectable after assessment by the thoracic surgeon.

**5.1.11 Required Laboratory Data:**

Granulocytes > 1.5 x 103 / L

Thrombocytes > 100 x 103 / L

AST, ALT, ALP < 2 times upper limit of normal

Creatinine clearance > 50 ml / min*

x Measurement or calculation based on gender from serum creatinine, age, and body weight (kg) according to the formula below:

For men: (140 - age) x (body weight in kg)

(72 x serum creatinine)

For women: 0.85 x (creatinine clearance)

**5.1.12 Required Diagnostic Procedures**

All patients must undergo esophagogastroduodenoscopy (EGD), CT scan of the chest and upper abdomen, CT scan of the head., Bone scan. or Positron emission tomography is recommended if available.

Bronchoscopy is required for patients with cancers adjacent to the trachea or left main stem bronchus.

Pulmonary function tests (PFTs) that include FEV1, DLCO/VA, and arterial blood gases.

**6.0 Screening and Randomization Phase**

**6.1 Recruitment**

Patients will be recruited from the referrals to either the London Health Sciences Centre or the London Regional Cancer Program. Potential patients may be referred to the study centre by local gastroenterologists, general internists, thoracic surgeons, medical or radiation oncologists or family physicians.

**6.2 Initial Screening**

All potentially eligible patients will be recorded (study logbook) with key demographic data such as name, address, sex, birth date, age, diagnosis and referring physician. A diagnosis of esophageal carcinoma must be established by the referring physician before the patient will be scheduled for an eligibility visit. All patients with potentially resectable esophageal carcinomas will be considered.

**6.3 Eligibility Visit Prior to Randomization**

Potential candidates will be seen by a surgical, medical, and radiation oncologist to be assessed for trial participation.

The following will be obtained at the eligibility visit: (1) written informed consent (2) history of relevant conditions and current medications (3) a physical examination including determination of patient's height, weight, and body surface area (4) baseline laboratory investigations including a PA and lateral chest radiographs; computed tomograms of chest and upper abdomen, computed tomograms of head, bone scan, pulmonary function tests, and arterial blood gases at rest. PET scans are also encouraged if available. Blood work including hemoglobin, total white cell count and differential, platelet count, sodium, potassium, glucose, creatinine, AST, ALP, bilirubin, albumin, LDH, calcium, magnesium. Baseline measures of health utility using the EuroQoL EQ-5D, and measures of quality of life using FACT-E and EORTC QLQ-C25 will be determined. Partial PAR (Patient Assessment Record) will be completed for baseline toxicity.

The eligibility visit will provide an opportunity for referral to other consultants to have medical therapy optimized. Randomization to treatment groups will follow a completed eligibility assessment. All patients eligible to be randomized will be recorded in a study log.

**7.0 Randomization**

With the completion of investigations and staging and the receipt of the baseline data recorded on the Eligibility Visit data collection forms the patient's eligibility will be re-checked and if the patient meets the inclusion and exclusion criteria, the patient will be randomized to one of two treatment groups. Blinded and blocked randomization will be in a 1:1 allocation ratio between treatment arms with stratification by surgeon. Randomization will occur centrally (Thoracic Clinical Trials Office) to ensure concealment of the process. Once randomized, the patient will be followed until death or the end of the study (1 year) and all outcome events will be attributed to the study group to which the patient has been assigned (intent-to-treat-analysis). Follow-up after 1 year will continue as per usual standard clinical practice. No clinical trial tests will be administered.

**7.1 Overview of Treatment Plan**

The control arm of this study will consist of induction chemotherapy (2 Cycles - Cisplatin plus continuous infusion of 5-Fluorouracil) and concurrent radiation followed by surgical resection of the esophagus. The experimental arm will consist of surgical resection followed by (if required based on final pathological staging) two cycles of chemotherapy (Epirubicin, Cisplatin and continuous infusion of 5-Fluorouracil) and concurrent extended volume radiation treatment with the last 2 cycles. Follow-up of patients with clinic visits of similar frequency to the experimental arm will be required to avoid introduction of bias in recording of time to progression.

**8.0 Surgical Treatment**

**8.1 Standardization of Technique**

All participating surgeons have agreed to follow a similar procedure. The patient's well-being and safety will always receive top priority and will be left to the individual surgeon's discretion and clinical judgement in the event of unexpected deterioration or emergency.

**8.2 Surgical Technique**

**8.2.1 Preoperative Evaluation**

All patients will undergo an evaluation to demonstrate no evidence of distant disease and no evidence of unresectable local regional invasion. A CT scan of the chest and upper abdomen, bone scan, CT scan of the head, and esophagogastroduodenoscopy are required at a minimum. A PET scan is encouraged if available and may replace a bone scan. Bronchoscopy will be done for mid esophageal tumours (< 25 cm from incisors on EGD) or if tracheobronchial invasion is suspected either clinically or on CT scan of the chest.

**8.2.2 Esophagectomy**

Esophagectomy will be performed using combined abdomen, right chest, and neck incisions for middle and upper third esophageal cancers. It is strongly recommended that the mediastinal pleura overlying the primary lesion and the mediastinal nodes and fat 5 cm proximal and distal to the primary lesion be excised en-bloc with the tumour.

Resection via the left chest or right chest and abdomen (Ivor-Lewis) may be done for mid-esophageal and gastroesophageal junction cancers. Transhiatal esophagectomy may be performed for lower third lesions. Our current standard is a minimally invasive approach (VATS and/or laparoscopic) as per tumour location and or surgeon preference.

The stomach is recommended for reconstruction, but a colon interposition may be used if technically appropriate. The anastomosis may be either stapled or sewn as per surgeon preference. A combined stapled and sewn anastomosis is recommended. Marking clips will be placed at the anastomosis if postoperative therapy is planned.

**8.2.3 Lymph Node Dissection**

At the time of esophagectomy, all lymph nodes will be removed if feasible, individually sampled and sent as separate specimens, labeled according the lymph node map. All technically accessible lymph nodes must be removed, rather than simply sampling a single representative node at each level.

**8.2.4 Proximal and Distal Margins**

Proximal and distal margins should be at least 2 cm beyond gross tumour as measured in the operating room after removal of the esophagus but prior to fixation of the specimen. Frozen sections should be obtained to ensure microscopically negative margins at the discretion of the operating surgeon. A microscopic proximal or distal margin < 1 mm will be considered positive, and the patient will remain eligible.

**8.2.5 Positive Resection Margins**

Patients that are found to have cancer positive proximal, or distal margins following preoperative chemoradiation and surgery should be considered for additional chemoradiation at the discretion of the medical and radiation oncologists. Patients in the experimental arm (postoperative chemoradiation) that were found to be T1-2 and N0 with positive resection margins should also be considered for postoperative chemoradiation.

**8.3 Surgical Quality Assurance**

**8.3.1 Completeness of Resection:** All pathology reports from patients in the study will be reviewed. Reports must contain information about gross and microscopic contamination of surgical resection margins. Tumour measurements must be recorded at surgical resection.

**8.3.2 Curative (R0):** Resections are defined as curative (complete resection: R0) when all gross disease has been removed, and microscopic examination reveals all surgical margins free of tumour, i.e., pathological stage T1-3, N0-1, M0 resected. Resections will still be considered curative if pathologic examination reveals positive lymph nodes if the gross nodes were completely resected.

**8.3.3 Palliative (R1 and R2):** Palliative resection (incomplete resection: R1 or R2) will be considered to have taken place when gross disease has been left behind (R2), or when microscopic examination reveals surgical margins which are not free of tumour (R1). Positive margins are defined as tumour at or less than 1 mm from the proximal or distal margins.

**8.3.4 No resection:** The primary tumour could not be removed.

**9.0 CHEMOTHERAPY (OPIS orders: QUINTETT-ADJ or QUINTETT–NEOADJ)**

**Arm I: Preoperative Chemotherapy (Control Arm) QUINTETT-NEOADJ**

Treatment should begin within 4 weeks of randomization consisting of 2 chemotherapy cycles concurrent with radiation. Cisplatin 25 mg/m2 days 1, 2, 3, and 4 concurrent with 5FU 1000 mg/m2 / day for 96 hours continuous venous infusion, starting with the beginning of radiation therapy. Chemotherapy should start simultaneously with the first day of radiation treatment if possible, but in no circumstance later than day 5 of radiation treatment, and not earlier than on the first radiation treatment day.

This regimen is repeated at week 5 of radiation therapy.

**Arm II: Postoperative Chemotherapy (Experimental Arm) QUINTETT-ADJ**

Treatment should begin between 8 to 12 weeks following surgery.

The chemotherapy treatment is as follows:

Chemotherapy alone for 2 cycles – One cycle consists of Epirubicin 50 mg/ m2 and Cisplatin 60 mg/m2 day 1, and 5-Fluorouracil (5FU) by continuous venous infusion at 200 mg/m2 for 21 days.

Chemotherapy (concurrent with radiation) immediately afterwards for 2 additional cycles – One cycle consists of Cisplatin 60 mg/m2 day 1, and 5-Fluorouracil (5FU) by continuous venous infusion at 200 mg/m2 for 21 days. Concurrent radiation is to start on the same day as cisplatin is administered if possible, but in all cases within 3 days before or after that date.

Details regarding the chemotherapeutic agents:

**9.1.1 Availability**

Cisplatin is commercially available as a sterile lyophilized powder in 10 mg and 50 mg vials when dissolved in 10 ml and 50 ml of sterile water respectively for injection (1 mg / ml). It is also available in an aqueous solution (Platinol-AQ7) with a concentration of 1 mg / ml in 50 and 100 mg vials. Do NOT use aluminum needles or aluminum containing IV sets. Further dilute the desired dose in a least 100 ml (500 ml to 1 liter is preferred) of 0.9% sodium chloride (NS) for infusion.

**9.1.2 Storage and Stability**

Unopened vials are stable for 2 years, Reconstituted vials are stable for 20 hours at room temperature, or for 72 hours if reconstituted with bacteriostatic water. DO NOT REFRIGERATE cisplatin solutions, as it will precipitate.

**9.1.3 Administration**

Preoperative chemotherapy: Cisplatin 25 mg/m2 bolus IV (in a 250 mL bag) infusion over 30 minutes on days 1, 2, 3, and 4 with prehydration, other standard renal-protective measures, and appropriate antiemetic coverage. The following is recommended:

Prior to cisplatin, begin intravenous hydration with 500 mL normal saline IV over 1 hour. Furosemide 40 mg IV in a 50 mL mini bag with hydration. After the cisplatin infusion, complete 250 ml of normal saline IV hydration over 30 minutes.

Postoperative chemotherapy: Cisplatin 60 mg/m2 bolus IV (in a 500 mL bag) infusion over 1 hour on days 1, 22, 43, and 64 with prehydration, other standard renal-protective measures, and appropriate antiemetic coverage. The following is recommended:

Prior to cisplatin, begin intravenous hydration with 1,000 mL normal saline IV over 2 hours. Furosemide 40 mg IV in a 50 mL mini bag with hydration. After the cisplatin infusion, complete 500 ml of normal saline IV hydration over 1 hour.

Anti-emetics include dexamethasone 4 mg po q12h starting the morning of the first dose of chemotherapy and continuing for 5 doses after the last cisplatin dose of each cycle; ondansetron 8 mg po q12h starting the morning of the first dose of chemotherapy and continuing for 5 doses after the last cisplatin dose of each cycle; prochlorperazine 10 mg po q4h prn.

The patient should be encouraged to drink as much liquid as possible overnight. Needles, syringes, catheters or IVC administration sets containing aluminum parts should not be used, as contact with cisplatin yields a black precipitate.

**9.1.4 Toxicities**

Cisplatin most commonly causes myelosuppression, nausea and vomiting, diarrhea, alopecia, renal toxicity, electrolyte wasting (e.g., potassium, magnesium, phosphate, calcium), peripheral neuropathies, and ototoxicity (high frequency hearing loss, tinnitus). Less commonly, hypersensitivity reactions, severe myalgias and neuropathies, optic neuritis, hyponatremia, elevated liver enzymes, and hyperuricemia may occur.

**9.1.5 Drug Interactions**

Concomitant administration with other nephrotoxic or ototoxic drugs, such as aminoglycosides, amphotericin B, furosemide, and methotrexate, increases the risk of renal failure and / or hearing loss. Decreased phenytoin levels have been observed when cisplatin is given with phenytoin therapy.

**9.2 5-Fluorouracil (5-FU, fluorouracil, Efudex7, Adrucil7)**

**9.2.1 Availability**

5-FU is commercially available and is an antimetabolite that interferes with RNA and DNA synthesis. Fluorouracil injection (Roche Laboratories): 50 mg / ml, 10 ml vials; clear, yellow, aqueous solution; Fluorouracil (Cetus, Lyphomed, Americal): 50 mg / ml; 10 ml, 20 ml, 100 ml vials, 10 ml ampules; Fluorouracil (Solopak): 50 mg / ml, 10 ml ampules.

**9.2.2 Preparation**

Inspect for precipitate; if found, agitate, or gently heat in water bath. Filter ampules with aspiration needle (5 μm). Compatible with D5W, 0.9% NS, D5LR.

Additive incompatibility: carboplatin, cisplatin, cytarabine, diazepam, doxorubicin, droperidol, epirubicin.

Y-site incompatibility: filgrastim, ondansetron, vinorelbine, dexamethasone.

**9.2.3 Storage and Stability**

Store at room temperature and protect from light. Dark yellow color indicates decomposition. Stable in polypropylene syringes. Stable in PVC reservoirs for infusion pump for 12 days. May adsorb to glass surfaces. Stable in cellulose nitrate / acetate ester or Teflon filters.

**9.2.4 Administration**

Postoperative chemotherapy: 5-Fluorouracil 200 mg / m2 / day continuously for 4 cycles of 21 days each, with cisplatin and epirubicin on days 1 and 22, and cisplatin on days 43 and 64 as indicated under 9.1 above. The CIV 5FU will thus continue for a total of 12 weeks, barring dose adjustments due to toxicities.

5-FU will be administered intravenously by continuous infusion through a Percutaneous Intravenous Central Catheter (PICC), or other central line, using a standard mechanical or electrical pump.

Preoperative chemotherapy: 5-Fluorouracil 1000 mg / m2 / day continuously for 4 days (96 hours) concurrently with radiation, and together with cisplatin daily (see above). This cycle is repeated starting with the 5th week of radiation therapy.

**9.2.5 Toxicity**

Nausea, vomiting (mild); ileus; diarrhea; stomatitis: 5-8 days after treatment initiation; gastric ulceration; myelosuppression: leukopenia, granulocytopenia (9-14 days); thrombocytopenia (7-14 days); alopecia; loss of nails; hyperpigmentation; photosensitivity; maculopapular rash; palmar-plantar erythrodysethesias: (42-82% receiving continuous infusion); CNS effects: disorientation, confusion (rare); cardiotoxicity; myocardial infarction, angina, asymptomatic S-T changes 68%; ocular effects (rare).

**9.2.6 Drug Interactions**

Leucovorin and other reduced folates enhance toxicity of 5-FU. Vitamins with large amounts of folic acid should be avoided.

**9.3 Epirubicin**

**9.3.1 Availability**

Epirubicin hydrochloride is commercially available as a 2 mg/ml solution for injection in 25 ml and 100 ml vials.

**9.3.2 Preparation**

Please refer to the package insert from the manufacturer for appropriate preparation techniques.

**9.3.3 Storage and Stability**

Intact vials should be stored under refrigeration between 2○ C and 8○ C. Epirubicin is reportedly stable in polypropylene syringes (or the original vial) for at least 14 days at room temperature and 180 days under refrigeration. However, the manufacturer recommends that the solution should be used within 24 hours of penetrating the rubber stopper.

**9.3.4 Administration**

Epirubicin 50 mg / m2 IV bolus will be given on days 1 and 22. Because of significant radiosensitizing toxicities of Epirubicin, the drug will be withheld during radiation treatment. Intravenous administration of Epirubicin should be performed with caution. It is recommended that Epirubicin be administered into the tubing of a freely flowing intravenous infusion (0.9% sodium chloride or 5% glucose solution) over a period of 3 to 15 minutes.

**9.3.5 Toxicity**

Potential toxicities include nausea, vomiting, diarrhea, mucositis, neutropenia, thrombocytopenia, anemia, alopecia, and more rarely cardiotoxicity and second malignancy such as acute leukemia. Necrosis at extravasation and radiation recall can also occur.

**9.4 Dose Modifications and Management of Toxicity**

Toxicity will be graded according to NCI Common Toxicity Criteria.

**9.4.1 Dose Modification for Obese Patients**

All dosing will be calculated according to the patient’s BSA as calculated from actual weight and height. Body surface area will be capped at 2.1 m2.

**9.4.2 Dose Modifications for Chemotherapy**

All dose modifications will be carried forward throughout the chemotherapy and chemoradiotherapy.

If a patient requires a dose reduction of a chemotherapy drug, that dose reduction will continue throughout subsequent chemotherapy and chemoradiotherapy. For example, if a patient requires a 25% reduction during cycle 1 (first cycle of ECF) that patient will receive 75% of the original dose throughout all subsequent cycles of therapy. Similarly, Cisplatin reductions will continue through all cycles. Epirubicin will not be given during cycles with concurrent radiation treatment.

Renal Toxicity.

If, on day 1 of cycles 2-4 the creatinine is greater than 1.5 times the upper limit of normal despite adequate hydration, a calculated creatinine clearance should be performed before that specific cycle is delivered. Based on the calculated creatinine clearance, subsequent dose reductions should be as follows:

If the creatinine clearance ≥ 80 ml/min, no dose reduction is required.

If the creatinine clearance is ≥ 60 and < 80 ml/min the dose of Cisplatinum will be reduced by 50% of the previous dose. The doses of 5-FU and Epirubicin will not change. The doses of Cisplatinum will not be re-escalated in later cycles.

If the creatinine clearance is < 60 ml/min, the patient will go off protocol therapy.

Peripheral Neuropathy.

For grade 0 or 1 peripheral neuropathy, no dose reduction is mandated.

For grade 2 neuropathy, the dose of Cisplatinum will be reduced to 75% of the previous dose. The doses of 5-FU and Epirubicin will not change. The doses of Cisplatinum will not be re-escalated in later cycles.

For grade 3 peripheral neuropathy, the patient should discontinue protocol therapy

Ototoxicity

Any patient developing grade 3 ototoxicity should discontinue protocol therapy.

Hepatic Dysfunction

Patients who develop a bilirubin of > 20 umol/L will have treatment interrupted for one week. Serum bilirubin will be rechecked the following week. If bilirubin is greater than 20 umol/L upon rechecking, treatment will be delayed a second week. If bilirubin remains over 20 umol/L after three weeks, discontinue protocol therapy. Epirubicin will be reduced to 50% of the previous dose for an AST of 2 to 4 times the ULN. Treatment should be held for an AST of > 4 times the ULN. If the AST levels do not resolve to baseline levels within 3 weeks, discontinue protocol therapy.

Cardiotoxicity

Patients developing signs of congestive heart failure should undergo evaluation of ventricular function. If studies demonstrate left ventricular ejection fraction of less than 50%, the patient should discontinue protocol therapy.

Dose Modifications for Stomatitis/Mucositis/Esophagitis/Diarrhea

All patients should be instructed in the appropriate diet and use of loperamide to control diarrhea secondary to chemotherapy, in accordance with the centre standards (for example, loperamide two tablets after each loose bowel movement to a total of 10 tablets per day). If diarrhea persists, or Stomatitis, mucositis or esophagitis occur the following actions should be taken:

*Stomatitis/Mucositis/Esophagitis/Diarrhea*

| CTC Grade at time when next cycle is due to start | Action | Dose of Drug (% previous dose) | | |
| --- | --- | --- | --- | --- |
|  |  | 5-FU | Epirubicin | Cisplatinum |
| 0 | Continue therapy | 100% | 100% | 100% |
| 1 | Continue therapy | 100% | 100% | 100% |
| 2 | Hold therapy for at least one week. Resume when toxicity ≤ 1 | 75% | 100% | 100% |
| 3 | Hold therapy for at least one week. Resume when toxicity ≤ 1 | 75% | 75% | 100% |
| 4 | Hold therapy for at least one week. Resume when toxicity ≤ 1 | 75% | 75% | 100% |

Doses of chemotherapy that have been reduced will not be re-escalated.

Dose Modification for Palmar-plantar erythrodysethesias

*Palmar-plantar erythrodysethesias (Hand-Foot-syndrome)*

| CTC Grade at time when next cycle is due to start | Action | Dose of Drug (% previous dose) | | |
| --- | --- | --- | --- | --- |
|  |  | 5-FU | Epirubicin (if applicable) | Cisplatinum |
| 0 | Continue therapy | 100% | 100% | 100% |
| 1 | Continue therapy and start pyridoxine 50 mg po TID | 100% | 100% | 100% |
| 2 | Hold therapy for at least one week. Resume when toxicity ≤ 1 Start pyridoxine 50 mg po TID | 75% | 100% | 100% |
| 3 | Hold therapy for at least one week. Resume when toxicity ≤ 1 Start pyridoxine 50 mg po TID | 75% | 100% | 100% |

Patients who do not recover to ≤ grade 1 within 3 weeks will discontinue protocol therapy.

Hematologic Toxicity

Patients whose platelet and/or neutrophil counts are low will have their therapy delayed (with weekly bloodwork) until adequate counts are achieved. The dose of chemotherapy used when therapy is resumed is adjusted as indicated in the following table (applies to all agents in a multi-agent regimen).

A new dose of chemotherapy should not be initiated (including the next seven days of a continuous 5-FU infusion in the postoperative arm) unless the absolute neutrophil count (ANC) is ≥ 1500/mm3 and the platelet count is ≥ 100,000/mm3.

Patients who experience neutropenia and do not recover to ANC ≥ 1500/mm3 within 3 weeks will discontinue protocol therapy.

Patients who experience thrombocytopenia and do not recover to platelet count ≥ 100,000/mm3 within 3 weeks will discontinue protocol therapy.

Dose to be given at next cycle (as percentage of the dose calculated based on body surface area), tabulated by worst myelosuppression experienced in previous cycle

| \| Dose to be given at next cycle (as percentage of the dose calculated based on body surface area), tabulated by *worst* myelosuppression experienced in *previous* cycle \| \| --- \| |
| --- | --- |
| \| Platelet Count (10^9^/L) \| Neutrophil or Granulocyte Count (10^9^/L) \| \| \| \| \| --- \| --- \| --- \| --- \| --- \| \|  \| > or = 1.5 \| 1 - 1.49 \| 0.5 - 0.99 \| < 0.5 \| \| > or = 100 \| 100% \| 100% \| 75% \| 50% \| \| 50-99 \| 100% \| 100% \| 75% \| 50% \| \| 25-49 \| 75% \| 75% \| 75% \| 50% \| \| < 25 \| 50% \| 50% \| 50% \| 50% \| |
| \|  \| \| --- \| |

**10.0 RADIATION THERAPY**

Chemoradiation will be offered to patients with either pT3 or pN1 resected tumours. Resected pT1N0, pT2N0 patients will not be offered adjuvant therapy but will be followed and analyzed together in the experimental arm using the intention-to-treat principle.

Patients that haveR1 or R2 positive resection margins following induction chemoradiation will not receive adjuvant chemotherapy.

The total dose for both arms will be 50.4Gy (1.8Gy/fx/day), 5 days a week, prescribed to the periphery (95% isodose curve of the PTV.)

External Beam Equipment

Megavoltage equipment is required with effective photon energies >_ 6MV.

Treatment Planning – 3 D.

Treatment planning CT study will be required to define gross tumor volume (GTV) and

planning target volume (PTV). For this study, local regional nodes (whether clinically

positive or negative) will be included in the clinical target volume (CTV). Each patient will be positioned in an individualized immobilization device in the treatment position on

a flat table. The GTV and PTV and normal organs will be outlined on all appropriate CT

slices and displayed using beam’s eye view. Normal tissues to be contoured included both lungs, skin, heart, spinal cord, esophagus, kidneys, and liver. 4D-CT is allowed if applicable. VQ SPECT is optional. Patient immobilization is required. Patient may be placed in the supine or prone position.

**Arm I: Preoperative Radiation Therapy (Control Arm)**

Radiation treatment will begin concurrent with chemotherapy cycle 1. Chemotherapy should start simultaneously with the first day of radiation treatment if possible, but in no circumstance later than day 5 of radiation treatment, and not earlier than on the first radiation treatment day.

The treatment planning target volume will be defined as per RTOG - 0113 with minimum modification. For mid-esophageal primaries (at or below the carina), the paraesophageal nodes need to be included-not the supraclavicular or celiac. For distal/gastroesophageal primaries, the field the celiac nodes should be included.

Barium swallow during the planning CT is optional provided a diagnostic chest was done with contrast to delineate the outline of the esophagus.

Target volumes

The Gross Tumor Volume (GTV) is defined as all known gross disease as defined by the planning CT and clinical information. Gross tumor includes the primary tumor (GTV-P) only. For the present protocol we have chosen to define the CTV a minimum of 4 cm proximal and distal and 1 cm lateral beyond the GTV delineated by CT scan and /or endoscopy. The final CTV may be larger for distal primaries since celiac nodes need to be included. The Planning Target Volume (PTV) will provide margin around the CTV to compensate for variability in treatment setup, breathing, or motion during treatment. The PTV volume must include 1cm around the CTV. The final superior and inferior margins will be approximately 5 cm beyond the GTV, and the lateral, anterior, and posterior margins will be approximately 2 cm beyond the GTV. If 4D-CT is used, IGTV (combinations of GTVs from full inspiration and expiration phases) should be determined. The IPTV will be 0.5cm around the ICTV.

Treatment Plan

3D Conformal Planning

Three-dimensional coplanar or non-coplanar beam geometry will be custom designed for each case to deliver highly conformal prescription dose distributions. The isocenter is defined as the common point of gantry and couch rotation for the treatment unit. The treatment plan will be composed of multiple static beams or arcs. The plan should be normalized to a defined point within the PTV. The PTV should be covered by the 95% isodose line with minimal hotspots (105%).

An example of a 3D-Conformal plan is to initially treat the PTV with a parallel opposed (AP/PA) geometry, followed by obliques to exclude the spinal cord.

Phase I 39.6Gy/ 22fx (1.8Gy/fx)

The superior and inferior margins of the PTV will be approximately 5 cm beyond the GTV, the lateral, anterior, and posterior margins will be approximately 2cm (except vertebral body posterior will be on the bony surface) beyond the GTV. For the distal primaries, the celiac nodes need to be included. Radiation fields arrangement can be APPA or multiple.

Phase II 10.8Gy/6fx (1.8Gy/fx)

Same PTV volume as in phase I but radiation fields will oblique off the spinal cord. Radiation field arrangement can be multiple to come off the cord and other critical organs.

Intensity Modulated Radiation Therapy (IMRT)

IMRT is allowed in this study and should be considered only when target coverage, OAR dose limits, or dose spillage are not achievable with 3D conformal planning. IMRT plans should follow the same planning principles as for 3D conformal planning. The number of segments (control points) and the area of each segment should be optimized to ensure deliverability and avoid complex beam fluences.

A single phase of 50.4 in 28 fractions (1.8 Gy. /fx) will be sufficient.

**Arm II: Postoperative Radiation Therapy (Experimental Arm)**

Radiation treatment will begin with chemotherapy cycle 3. Concurrent radiation is to start on the same day as cisplatin is administered if possible, but in all cases within 3 days before or after that date.

Treatment planning target volume as per Yu, et al, (26) and Yu, et al (27), with minimum modification.

Target volumes

For the present protocol in post –op patients there is no GTV unless with residual disease and it should be delineated with surgical clips. GTV-P is defined as pre-op gross tumor volume as above. CTV should include the tumor bed and the anastomosis. The CTV will be 1 cm proximal of the anastomosis, 4 cm distal and 1 cm lateral, anterior, posterior beyond the pre-op GTV-P delineated by CT scan and /or endoscopy. For distal primaries celiac nodes (around T12 L1) should be included in the treatment fields.

Planning Target Volume (PTV) will provide margin around the CTV to compensate for variability in treatment set up, breathing, or motion during treatment. A margin of 1 cm around the CTV will define the PTV.

The final PTV superior margin will include the surgical anastomotic site (labeled with radio-opaque clips) proximally with 2 cm. The inferior margin of the field will be 5 cm beyond the pre-op GTV-P location. Lateral, anterior, and posterior borders will be 2 cm beyond the borders of the tumor bed and regional lymph nodes. Peri-esophageal lymph nodes will be included. A barium swallow or radio - contrast such as Esophocat® (preferred) may be obtained at the time of simulation to confirm the post -op location of the esophagus and stomach.

Treatment Plan

3D Conformal Planning

Phase I 30.6Gy/17fx (1.8Gy/fx)

The superior margin of the PTV will include the surgical anastomotic site (labelled with radio-opaque clips) proximally with 2 cm. The inferior margin of the field will be 5 cm beyond the previous GTV-P location. Lateral, anterior, and posterior borders will be 2 cm (except vertebral body posterior will be on the bony surface) beyond the lateral borders of the tumor bed and regional lymph nodes. For the distal primaries, the celiac nodes (around T12 L1) need to be included. Peri-esophageal lymph nodes will be included. Radiation fields arrangement can be APPA or multiple.

Phase II 19.8Gy /11fx (1.8Gy/fx)

This is the coned down field to include the pre-op GTV-P bed only. The PTV-P is 5 cm proximal and distal from pre-op GTV-P. The lateral, anterior, and posterior margins will be approximately 2 cm (except vertebral body posterior will be on the bony surface) beyond the pre-op GTV-P.

Radiation fields arrangement can be multiple to come off the cord and other critical organs.

The surgical anastomotic site should receive 30.6 Gy in 17 fractions if it is not involved with disease. It should be boosted to a maximum total dose of 50.40 Gy in 28 fractions if it is close or positive margins (together either within or outside the preop GTV-P at the discretion of the treating physician).

IMRT Planning

IMRT treatment planning is permitted. Should IMRT be the treatment planning choice, 36-38 Gy/ 28 fx is to deliver to include the anastomosis and 50.4Gy/28fx is to deliver to include the tumor bed (PTV-P) only.

Normal Tissue Volume and Tolerances.

The following organs and doses by volume are guidelines for the 3D treatment plan. All normal tissues assume treatment at 1.8 Gy/fx.

Lung V20 <30% (TL-GTV), V20 <35% (TL-PTV)

Spinal Cord 10cm < 46Gy; 20cm <40Gy

Heart 1/3< 45Gy, 2/3< 40Gy, 3/3< 35Gy

Liver 1/3<40Gy, 2/3<30Gy, 3/3< 25Gy

Kidney 1/3 < 40Gy, 2/3 < 25 Gy, 3/3 < 15 Gy

Stomach 1/3< 60Gy, 2/3 < 50 Gy, 3/3 < 45Gy

Esophagus 1/3 < 60Gy, 2/3< 55Gy, 3/3 < 50Gy

Medical physicist, Dr. Stewart Gaede may be contacted for additional information regarding radiation planning techniques and resources.

Therapy Interruptions

If interruption of therapy (up to two weeks) becomes necessary, radiation therapy should be completed to the prescribed doses. Total number of fractions and elapsed days should be carefully reported. If an interruption of more than 2 weeks is necessary, resumption of treatment is at the discretion of the radiation oncology chair. The patient will be considered a major deviation, but follow-up will be continued.

Criteria for Toxicity.

Acute and late toxicity related to radiation therapy include fatigue, myelosuppression, skin erythema, subcutaneous fibrosis, esophagitis, carditis, myelitis, acute radiation pneumonitis and late pulmonary fibrosis, and esophageal stricture. Acute toxicity monitoring: Acute (<- 90 days from RT start) side effects of radiation therapy will be documented using the revised NCI Common Toxicity Criteria, version 3.0 (any more recent ones?).

Later toxicity monitoring. Late (> 90 days since RT start or persisting beyond 90 days) post-treatment complication will be evaluated and graded according to the RTOG Late Effect Radiation Morbidity Criteria.

**11.0 Outcomes**

**11.1 Primary Outcome**

**Quality-of-Life** Quality-of-Life as measured by the FACT-E over 1 year from randomization.

**11.2 Secondary Outcomes**

**11.2.1 Survival at 5 years.** All patients will be followed every 3 months for 2 years and then every 6 months up to 5 years or until death if it occurs before 5 years. Survival will be measured from randomization.

**11.2.2 Disease Free Survival.** Recurrence rates in the two groups including local (gastric or esophageal bed), regional (regional lymph nodes), and distant recurrences (supraclavicular lymph node, liver, peritoneal carcinomatosis, or lung, brain, etc.) will be compared. Disease free survival will be measured as the time from patient entry onto study until the first malignant disease recurrence.

**11.2.3 Site of Treatment Failure.** The location of the first site of malignant disease recurrence will be recorded. Local failure occurs within the gastric or esophageal bed, or regional lymph nodes. Distant failure occurs in the supraclavicular lymph nodes, liver, peritoneal carcinomatosis, pleura, lung, bones, brain, or elsewhere not considered local.

**11.2.4 Toxicities**. Possible adverse drug or radiation events will be reported as described in the London Regional Cancer Program Patient Assessment Record (PAR).

**11.2.5 Completeness of Resection.** The number of R0, R1, R2, and unresectable patients will be compared.

**11.2.6 Economic Evaluation.** Within the clinical trial we will also conduct an economic comparison of the two treatments. In summary, the viewpoint of the economic study will be that of a Provincial Ministry of Health and we will therefore document all direct health care costs associated with the treatment strategies over the 5-year period of follow-up. Case report forms will be designed to document physical items of resources consumed (i.e., tests, procedures, hospital stay) and unit prices will be estimated for resources using cost-accounting methods. In a circumstance where one of the tri-modality therapies is both more costly and more effective (i.e., greater number of QALYs) we will calculate the incremental cost per QALYs as the ratio of the difference (treatment minus control) in cost to the difference in QALYs. Incremental cost per QALYs estimates will be presented with associated 95% confidence intervals estimated by a resampling technique known as bootstrapping. To further explore uncertainties in the data and analysis we will also conduct extensive sensitivity analyses. We will attempt to project, using available data, costs, and outcomes to 5 years to ascertain whether the economic appraisal is sensitive to the time horizon of analysis.

**12.0 Follow-up and Data Collection**

Every effort will be made to make the outcome assessments as objective as possible as it is not possible to blind patients to the treatment arm to which the patient had been allocated.

**12.1 Follow-up Schedule**

The follow-up visits will occur at the discretion of the treating surgical, medical, and radiation oncologists. All patients will be followed for events until 60 months or death. Routine care will include a history and physical and a chest radiograph. Quality-of-Life, toxicities and morbidity data will be completed only during the first year following randomization at baseline, and every 2 months up to 1 year.

**12.2 Special Event Data**

A Special Event form will require completion for every hospitalization and death. The primary and secondary diagnosis for each hospitalization will be recorded in patients who suffer an adverse event. Detailed information will be collected for the death of every patient including the review of death certificates.

**13.0 Central Adjudication of all Events**

All suspected treatment related morbid events or deaths will be reviewed blind to the treatment group by the Events Adjudication Committee. This will be done every 6 months for the following events: (1) death classified by cause; (2) toxicities related to chemotherapy, radiation, or surgery; (3) all hospitalizations.

**14.0 Adverse Events and Safety**

**14.1 Adverse Event Reporting**

Any fatal (grade 5) or life-threatening (grade 4) adverse reaction that is due to or suspected to be the result of protocol treatment must be reported to the Clinical Trials Group Chairperson by telephone within 24 hours of discovery. Unknown adverse reactions (> grade 2) must be reported to Clinical Trials Group and the local IRB within 10 working days of discovery. Reactions thought not to be treatment related should not be reported; however, a report should be made if there is a reasonable suspicion that the effect is due to protocol treatment.

**15.0 Management of Intercurrent Events**

Several illnesses and major events may befall patients during the study. Referring physicians are free to treat each patient according to their best judgement. However, when in doubt they are encouraged to discuss an individual's management with the Thoracic Clinical Trials Centre.

The clinical course of esophageal cancer one in which a significant number of patients treated with standard therapy will deteriorate or die. Significant deterioration may be in the form of worsening symptoms of dysphagia, pain, anorexia, weight loss due to disease progression. The treating oncologist should always use the most appropriate management that is in the best interest of the patient.

**15.1 Ancillary Therapy**

Patients should receive full supportive care, including transfusions of blood and blood products, erythropoietin, antibiotics, antiemetics, etc., when appropriate. Filgrastim (G-CSF) and sargramostim (GM-CSF) treatment is discouraged. They may not be used prophylactically to avoid dose reductions or delays.

**15.2 Other Agents**

Treatment with hormones or other chemotherapeutic agents may not be administered except for steroids given for adrenal failure; hormones for non-disease related conditions (e.g., insulin for diabetes); and intermittent use of dexamethasone as an antiemetic.

**15.3 Nutrition**

All patients undergoing preoperative chemoradiation (Control Arm) will undergo the insertion of a percutaneous feeding gastrojejunostomy for enteral nutritional support. Patients undergoing postoperative chemoradiation will have a surgically placed feeding jejunostomy for supplemental nutrition as needed.

**16.0 Participant Safety and Confidentiality**

The study physicians will be responsible for the safety or participants under his or her care. The Steering Committee will have primary responsibility for the monitoring of study data for adverse trends and morbidity. The confidentiality of all participants will be protected both at the study site and the Study Coordinating and Methods Center. Completed data collection forms will receive the same protection as other medical records. Reporting of study results will not identify individual patients.

**17.0 Sample Size Determination**

**17.1 Sample Size**

The sample size is based on an improvement in the quality-of-life as measured by the FACT-E at one year. If we assume an improvement in the treatment arm of 15 points, 2- sided α=0.05, β= 0.2 and σ=25 (28)

Formula:

n per group = (Z_α_ + Z_β_)^2^ *2σ^2^

Δ^2^

σ=25

Δ=15

n=44 per arm.

**Summary of Sample Size Estimates**

| **σ** | **Δ** | **α** | **β** | **n**  **(each arm)** | **Add 10%** | **Total N** |
| --- | --- | --- | --- | --- | --- | --- |
|  |  |  |  |  |  |  |
| 25 | 10 | 0.05 | 0.2 | 98 | 108 | 216 |
| 25 | 15 | 0.05 | 0.1 | 58 | 64 | 128 |
| **25** | **15** | **0.05** | **0.2** | **44** | **48** | **96** |
| 25 | 20 | 0.05 | 0.1 | 33 | 36 | 72 |
| 25 | 20 | 0.05 | 0.2 | 25 | 27 | 54 |
| 25 | 25 | 0.05 | 0.1 | 21 | 23 | 46 |
| 25 | 25 | 0.05 | 0.2 | 16 | 17 | 34 |

The sample size was estimated based on an improvement in the HRQOL as measured by the FACT-E at one year of 15 points in the Neoadjuvant arm compared to the Adjuvant arm. Based on a two-sample t-test, using 2-sided testing, alpha = 0.05, power = 80% and standard deviation = 25, 48 patients would be required in each arm (96 total patients) after adjusting for 10% lost to follow-up (32). The randomization was completed according to a computer-generated randomization list by an independent statistician who had no clinical involvement in the trial and then allocated by the Thoracic Clinical Trials office to ensure concealment of the randomization process (33). Patients were randomized in a 1:1 allocation ratio and stratified by nodal status and surgeon.

**17.2 Statistical Analysis**

Descriptive statistics were generated for all patients and stratified by treatment arm for baseline patient, tumour and treatment characteristics and stratified by treatment arm for HRQOL endpoints stratified by follow-up visit, compared using the chi-square test, Fisher’s exact test, two-sample t-test, Wilcoxon rank sum test or paired t-test (compared to baseline only) as appropriate. Univariable Cox proportional hazards regression was performed for OS and DFS for all eligible variables for all patients (Supplemental Table 1 online). Kaplan-Meier estimates were generated for OS and DFS for all patients and stratified by treatment arm compared using the stratified log-rank test stratified by clinical nodal status (N0, N1). All statistical analysis was intention-to-treat and performed using SAS version 9.4 software (SAS Institute, Cary, NC, USA) using two-sided statistical testing at the 0.05 significance level. The trial was not powered to detect differences in the secondary outcomes including survival. There was no imputing, deletion, guessing, or substitution used for missing values. There were no interim analyses. There was no blinding of patients, investigators, or data analysts to the treatment allocation.

**17.3 Data Monitoring**

In case the trial has a dramatic effect early in the study, the investigator team has agreed to provide an interim analysis when the first 50 patients have passed the one year follow up, and if there is either a benefit, or a serious worsening of one group relative to the other. The data management centre will do the interim analysis and share it with the Safety Monitoring Committee to decide whether to stop the trial earlier than planned. While many may suggest that some adjustment for an interim analysis is needed; Pocock (51) and an article (52) have suggested that this is not necessary when the number of interim analyses is few (less than 2); and the level of interim analysis is at most 1%. Hence we propose the Type 1 error criterion for stopping early will be 1%, with no adjustment of the final alpha level if the trial is not stopped early (28,29).

**18.0 Trial Organization**

The proposed trial will be conducted in London, Ontario. Ottawa and Vancouver have expressed interest and may join. Policy decisions relating to the trial will be decided by the Steering Committee. The day-to-day coordination and central randomization will be provided by the Thoracic Clinical Trials Office at the London Health Sciences Centre, Victoria Hospital, London, Ontario.

**19.0 Publications**

The main publications for the trial will be in the names of all full collaborating investigators. Subsidiary papers will be authored by study investigators.

**20.0 Feasibility of Recruitment**

Pilot work from London has demonstrated that the procedure can be done safely with encouraging results (10). Approximately 50 esophagectomies occur in London each year. If London is the only centre, it will take two years to accrue the required number of patients.

**21.0 Limitations, Potential Pitfalls and Strategies**

Assessment bias always occurs in oncology trials because it is often impossible and impractical to blind the outcome assessment. This bias that may occur with quality-of-life instruments but can be minimized by using the self-administrated instruments that makes physician and data technician blinding unnecessary. This concern is not a problem when survival is a secondary outcome, as it is in this trial. The other outcomes could be blinded but are secondary questions and will be used for hypothesis generating. The added cost is not justified. Selection bias will be minimized by randomization just after the eligibility visit. All eligible, non-randomized patients will be recorded and used later in sensitivity analyzes. Crossovers between treatment arms will not be allowed.

New information may become available supporting combined modality therapy prior to completion of the trial. This may make it unethical to continue with the protocol. It would be unusual for clinical practice to drastically change based on the results of one or maybe two trials. Confirming evidence is always needed to support the results. We are not aware of any trial currently evaluating preoperative versus postoperative chemoradiation for resectable esophageal cancer.

Technical advances in the field, such as newer approaches to surgery, radiation, and newer chemotherapeutic agents could make the current approach archaic before the trial is completed, but this is very unlikely. The London surgeons are already performing minimally invasive esophagectomies and are the current leaders in Canada. The participating surgeons have agreed on the proposed surgical technique, are comfortable and have experience with it. We anticipate that it will remain the standard for at least a decade. Minor changes in the field will be incorporated into the procedure at a national level to remain current and provide state-of-the-art care.

**Study Schema**

**Esophageal Cancer**

Biopsy Proven

**Eligibility and Staging**

CT chest and upper abdomen

CT head

PFTs

Bone scan

**Assessment**

Surgical, Medical, and Radiation Oncologists

**Postoperative Chemoradiation**

Cisplatin + 5FU + Epirubicin

Extended Field Radiation

**Surgery**

**Preoperative Chemoradiation**

Cisplatin + 5FU

Radiation

T3 and/or N1

N0

**Surgery**

**Positive Resection Margins**

**Consider Chemoradiation**

**Clear Resection Margins**

Follow-up

**Randomization**

Stratified by Nodal status and Surgeon

**References**

1. Earlam R. An MRC prospective randomised trial of radiotherapy versus surgery for operable squamous cell carcinoma of the oesophagus [see comments]. Ann R Coll Surg Engl. 1991 Jan;73(1):8–12.

2. Blot WJ, Devesa SS, Kneller RW, Fraumeni Jr JF. Rising incidence of adenocarcinoma of the esophagus and gastric cardia [see comments]. J Am Med Assoc. 1991 Mar 13;265(10):1287–9.

3. Coia LR, Engstrom PF, Paul AR, Stafford PM, Hanks GE. Long-term results of infusional 5-FU, mitomycin-C and radiation as primary management of esophageal carcinoma. Int J Radiat Oncol Biol Phys JID - 7603616. 1991 Jan;20(1):29–36.

4. Malthaner R, Wong RKS, Spithoff K. Preoperative or postoperative therapy for resectable oesophageal cancer: An updated practice guideline. Clin Oncol. 2010;22(4):250–6.

5. Gebski V, Burmeister B, Smithers BM, Foo K, Zalcberg J, Simes J. Survival benefits from neoadjuvant chemoradiotherapy or chemotherapy in oesophageal carcinoma: a meta-analysis. Lancet Oncol. 2007;8(3):226–34.

6. Thirion S.;Le Maitre,A.;Tierney,J.; PG ;Michiel. Individual patient data-based meta-analysis assessing pre-operative chemotherapy in resectable oesophageal carcinoma. J Clin Oncol. 2007;25(Suppl 18):4512.

7. Macdonald JS, Smalley SR, Benedetti J, Hundahl SA, Estes NC, Stemmermann GN, et al. Chemoradiotherapy after surgery compared with surgery alone for adenocarcinoma of the stomach or gastroesophageal junction. N Engl J Med. 2001 Sep 6;345(0028–4793):725–30.

8. Earle CC, Maroun J, Zuraw L. Neoadjuvant or Adjuvant Therapy for Resectable Gastric Cancer Practice Guideline Report # 2-14. 2003;(December 2000).

9. Saito T, Shigemitsu Y, Kinoshita T, Shimoda K, Abe T, Nakamura A, et al. Cisplatin, vindesine, pepleomycin and concurrent radiation therapy following esophagectomy with lymph adenectomy for patients with an esophageal carcinoma 1965. Oncology. 1993 Jul;50(0030-2414 (Print)):293–7.

10. Bedard EL, Inculet RI, Malthaner RA, Brecevic E, Vincent M, Dar R. The role of surgery and postoperative chemoradiation therapy in patients with lymph node positive esophageal carcinoma. Cancer. 2001 Jun 15;91(12):2423–30.

11. Rice TW, Adelstein DJ, Chidel MA, Rybicki LA, DeCamp MM, Murthy SC, et al. Benefit of postoperative adjuvant chemoradiotherapy in locoregionally advanced esophageal carcinoma. J Thorac Cardiovasc Surg. 2003;126(5):1590–6.

12. Liu HC, Hung SK, Huang CJ, Chen CC, Chen MJ, Chang CC, et al. Esophagectomy for locally advanced esophageal cancer, followed by chemoradiotherapy and adjuvant chemotherapy 1591. World JGastroenterol. 2005 Sep 14;11(1007-9327 (Print)):5367–72.

13. Yu E, Dar R, Rodrigues GB, Stitt L, Videtic GM, Truong P, et al. Is extended volume external beam radiation therapy covering the anastomotic site beneficial in post-esophagectomy high risk patients? Radiother Oncol. 2004 Nov;73(0167-8140 (Print)):141–8.

14. Cella DF, Tulsky DS, Gray G, Sarafian B, Linn E, Bonomi A, et al. The Functional Assessment of Cancer Therapy scale: development and validation of the general measure. J Clin Oncol JID - 8309333. 1993 Mar;11(3):570–9.

15. Winstead-Fry P, Schultz A. Psychometric analysis of the Functional Assessment of Cancer Therapy-General (FACT-G) scale in a rural sample. Cancer JID - 0374236. 1997 Jun 15;79(12):2446–52.

16. Overcash J, Extermann M, Parr J, Perry J, Balducci L. Validity and reliability of the FACT-G scale for use in the older person with cancer. Am J Clin Oncol JID - 8207754. 2001 Dec;24(6):591–6.

17. Darling G, Eton DT, Sulman J, Casson AG, Celia D. Validation of the functional assessment of cancer therapy esophageal cancer subscale 1978. Cancer. 2006 Aug 15;107(0008-543X (Print)):854–63.

18. Lagergren P, Fayers P, Conroy T, Stein HJ, Sezer O, Hardwick R, et al. Clinical and psychometric validation of a questionnaire module, the EORTC QLQ-OG25, to assess health-related quality of life in patients with cancer of the oesophagus, the oesophago-gastric junction and the stomach 1972. EurJ Cancer. 2007 Sep;43(0959-8049 (Print)):2066–73.

19. Blazeby JM, Kavadas V, Vickery CW, Greenwood R, Berrisford RG, Alderson D. A prospective comparison of quality of life measures for patients with esophageal cancer 8. QualLife Res. 2005 Mar;14(0962-9343 (Print)):387–93.

20. Group E. EuroQol--a new facility for the measurement of health-related quality of life. The EuroQol Group. Health Policy (New York). 1990 Dec;16(0168-8510 (Print)):199–208.

21. Brooks R. EuroQol: the current state of play. Health Policy (New York). 1996 Jul;37(0168-8510 (Print)):53–72.

22. Rabin R, de CF. EQ-5D: a measure of health status from the EuroQol Group 1. AnnMed. 2001 Jul;33(0785-3890 (Print)):337–43.

23. Gold M. Panel on cost-effectiveness in health and medicine 1. Med Care. 1996 Dec;34(0025-7079 (Print)):DS197–9.

24. Gold MR, Seigel JE, Russel LB, Weinstein MC. Cost-Effectiveness in Health and Medicine. Vol. 1st. New York, NY: Oxford University Press Inc.; 1996.

25. Pickard AS, Neary MP, Cella D. Estimation of minimally important differences in EQ-5D utility and VAS scores in cancer 1. Heal QualLife Outcomes. 2007;5(1477-7525 (Electronic)):70.

26. Yu EW, Tai P, Rodrigues G, Ash R, Stitt L, Dar R, et al. Extended versus small field irradiation in high risk post esophagectomy patients receiving combined chemoradiation therapy: A decade experience in treatment of esophageal cancer. Radiother Oncol. 2005 Sep 1;76(Supplement 1):S45.

27. Yu E, Tai P, Younus J, Malthaner R, Truong P, Stitt L, et al. Postoperative extended-volume external-beam radiation therapy in high-risk esophageal cancer patients: A prospective experience. Curr Oncol. 2009;16(4):48–54.

28. Pocock SJ. The size of a clinical trial . In: Clinical trials: a practical approach . Toronto : John Wiley and Sons ; 1993. p. 123–41.

29. Interim analysis in the pharmaceutical industry. PMA Biostatistics and Medical Ad Hoc Committee on Interim Analysis. Control Clin Trials JID - 8006242. 1993 Apr;14(2):160–73.
