# Supplementary material for: The quality of life in neoadjuvant versus adjuvant therapy of esophageal cancer treatment trial (QUINTETT): Randomized parallel clinical superiority trial
Source: Thorac Cancer. 2022 May 24;13(13):1898–915. doi: 10.1111/1759-7714.14433 (PMC9250846; doi:10.1111/1759-7714.14433)
Supplement: Supplementary file 2 — Table S1 [file TCA-13-1898-s003.docx]

| **Supplemental Table 1. Univariable Cox proportional hazards regression models for all patients stratified by clinical nodal status and adjusted for treatment arm (n=96).** | | | | |
| --- | --- | --- | --- | --- |
| **Dependent Variable:** | **Overall Survival** | | **Disease-Free Survival** | |
| **Variable:** | **HR (95% CI)** | **p-value** | **HR (95% CI)** | **p-value** |
| **Univariable:** | | | | |
| **Adjuvant vs. neoadjuvant CRT** | 1.22 (0.76, 1.97) | 0.409 | 1.09 (0.69, 1.74) | 0.711 |
| **Age (per 5 years)** | 1.07 (0.92, 1.23) | 0.395 | 1.09 (0.94, 1.26) | 0.240 |
| **Male vs. female** | 1.23 (0.64, 2.35) | 0.533 | 1.28 (0.67, 2.44) | 0.452 |
| **Clinical T stage**  T2 vs. T1  T3 vs. T1 | 2.46 (0.91, 6.66)  3.11 (1.20, 8.06) | 0.063  0.078  **0.020** | 2.34 (0.94, 5.87)  2.44 (1.01, 5.90) | 0.133  0.069  **0.048** |
| **SUV_Max_ (per 5 units)** | 0.98 (0.81, 1.17) | 0.800 | 0.92 (0.76, 1.11) | 0.363 |
| **Chemotherapy (yes vs. no)** | 1.42 (0.59, 3.45) | 0.436 | 1.19 (0.52, 2.74) | 0.677 |
| **Chemotherapy per protocol (yes vs. no)** | 0.51 (0.28, 0.94) | **0.031** | 0.58 (0.32, 1.03) | 0.065 |
| **Chemotherapy modified (yes vs. no)** | 1.08 (0.66, 1.76) | 0.765 | 0.97 (0.60, 1.56) | 0.888 |
| **Chemotherapy stopped (yes vs. no)** | 1.23 (0.70, 2.16) | 0.464 | 1.13 (0.65, 1.97) | 0.658 |
| **Chemotherapy modified or stopped (yes vs. no)** | 1.49 (0.90, 2.47) | 0.120 | 1.30 (0.80, 2.13) | 0.292 |
| **Chemotherapy modified, stopped or other (yes vs. no)** | 1.88 (1.11, 3.17) | **0.019** | 1.62 (0.98, 2.68) | 0.061 |
| **Chemotherapy completed ≥ 1 cycle (yes vs. no)** | 0.93 (0.45, 1.89) | 0.833 | 0.83 (0.42, 1.65) | 0.592 |
| **Chemotherapy cycles (per 1 cycle)** | 0.89 (0.74, 1.06) | 0.195 | 0.87 (0.72, 1.04) | 0.116 |
| **Cisplatin (yes vs. no)** | 0.83 (0.40, 1.74) | 0.625 | 0.73 (0.36, 1.48) | 0.381 |
| **Cisplatin infusions (per 1 infusion)** | 0.85 (0.73, 1.01) | 0.058 | 0.83 (0.70, 0.98) | **0.024** |
| **Cisplatin dose reduced (yes vs. no)** | 1.12 (0.67, 1.86) | 0.664 | 1.02 (0.62, 1.69) | 0.934 |
| **5-Fluorouracil (yes vs. no)** | 0.80 (0.39, 1.63) | 0.536 | 0.72 (0.36, 1.42) | 0.339 |
| **5-Fluorouracil infusions (per 1 infusion)** | 0.97 (0.91, 1.03) | 0.361 | 0.96 (0.91, 1.02) | 0.225 |
| **5-Fluorouracil dose reduced (yes vs. no)** | 1.19 (0.73, 1.94) | 0.477 | 1.07 (0.67, 1.74) | 0.770 |
| **Epirubicin (yes vs. no)** | 0.97 (0.46, 2.04) | 0.942 | 0.85 (0.42, 1.74) | 0.659 |
| **Epirubicin infusions (per 1 infusion)** | 0.98 (0.71, 1.34) | 0.877 | 0.92 (0.68, 1.26) | 0.615 |
| **Epirubicin dose reduced (yes vs. no)** | 0.86 (0.38, 1.96) | 0.717 | 0.85 (0.37, 1.94) | 0.699 |
| **Radiotherapy (yes vs. no)** | 1.00 (0.47, 2.13) | > 0.99 | 0.88 (0.43, 1.82) | 0.727 |
| **Radiotherapy completed (yes vs. no)** | 0.35 (0.17, 0.75) | **0.006** | 0.39 (0.18, 0.80) | **0.011** |
| **Feeding tube inserted: Any (yes vs. no)** | 0.57 (0.20, 1.65) | 0.302 | 0.52 (0.20, 1.37) | 0.186 |
| **Feeding tube inserted: CRT (yes vs. no)** | 1.51 (0.86, 2.66) | 0.156 | 1.56 (0.90, 2.70) | 0.114 |
| **Feeding tube inserted: Before surgery (yes vs. no)** | 1.08 (0.52, 2.26) | 0.829 | 1.16 (0.57, 2.35) | 0.691 |
| **Feeding tube inserted: Post-operative stay (yes vs. no)** | 0.42 (0.22, 0.82) | **0.011** | 0.37 (0.19, 0.71) | **0.003** |
| **Feeding tube inserted: 1-month post-discharge (yes vs. no)** | 1.28 (0.70, 2.34) | 0.430 | 1.30 (0.73, 2.33) | 0.379 |
| **Surgery (yes vs. no)** | 0.17 (0.06, 0.44) | **< 0.001** | 0.08 (0.03, 0.22) | **< 0.001** |
| **Unresectable (yes vs. no)** | 6.36 (2.55, 15.84) | **< 0.001** | 22.41 (7.16, 70.13) | **< 0.001** |
| **Pathology (yes vs. no)** | 0.17 (0.08, 0.38) | **< 0.001** | 0.04 (0.02, 0.11) | **< 0.001** |
| **Pathological T stage**  T2-T4 vs. T0-T1 | 2.00 (1.04, 3.85) | **0.038** | 1.60 (0.86, 2.97) | 0.138 |
| **Pathological N stage**  N1 vs. N0  N2 vs. N0  N3 vs. N0 | 1.85 (0.94, 3.63)  2.17 (0.81, 5.81)  5.06 (2.09, 12.28) | **0.005**  0.075  0.123  **< 0.001** | 1.52 (0.79, 2.92)  1.83 (0.70, 4.78)  3.92 (1.67, 9.20) | **0.020**  0.211  0.217  **0.002** |
| **Histology**  Squamous vs. adenocarcinoma  Unknown vs. adenocarcinoma | 0.98 (0.40, 2.42)  10.42 (2.79, 38.96) | **0.002**  0.968  **< 0.001** | 0.98 (0.40, 2.41)  9.97 (2.71, 36.69) | **0.003**  0.973  **< 0.001** |
| **Tumour grade**  II vs. I  III vs. I | 1.85 (0.64, 5.34)  2.22 (0.75, 6.54) | 0.348  0.253  0.148 | 1.43 (0.55, 3.73)  1.83 (0.68, 4.89) | 0.438  0.467  0.229 |
| **Positive margins (yes vs. no)** | 2.08 (1.13, 3.82) | **0.019** | 2.35 (1.25, 4.41) | **0.008** |
| **Resection status**  R1 (“microscopic/margin positive”) vs. R0 (“complete”)  R2 (“unresectable”) vs. R0 (“complete”) | 1.93 (1.06, 3.51)  7.50 (2.95, 19.05) | **< 0.001**  **0.032**  **< 0.001** | 2.29 (1.23, 4.27)  29.99 (9.34, 96.28) | **< 0.001**  **0.009**  **< 0.001** |

**HR** – Hazard ratio; **CI** – Confidence interval; **NR** – Not reported; **CRT** – Chemoradiotherapy;

**SUV_Max_** – Maximum standardized uptake values; **CTC** – Circulating tumour cells; P-values < 0.05 shown in **BOLD**;

| **Supplemental Table 1 (Continued). Univariable Cox proportional hazards regression models for all patients stratified by clinical nodal status and adjusted for treatment arm (n=96).** | | | | |
| --- | --- | --- | --- | --- |
| **Dependent Variable:** | **Overall Survival** | | **Disease-Free Survival** | |
| **Variable:** | **HR (95% CI)** | **p-value** | **HR (95% CI)** | **p-value** |
| **Univariable:** | | | | |
| **Positive nodes (per 10%)** | 1.22 (1.10, 1.35) | **< 0.001** | 1.18 (1.07, 1.31) | **0.001** |
| **Any invasion (yes vs. no)** | 1.59 (0.84, 3.04) | 0.158 | 1.69 (0.90, 3.17) | 0.103 |
| **Stomach extension (yes vs. no)** | 1.42 (0.82, 2.48) | 0.211 | 1.68 (0.97, 2.89) | 0.063 |
| **Lymphovascular invasion (yes vs. no)** | 1.30 (0.69, 2.45) | 0.417 | 1.11 (0.60, 2.04) | 0.738 |
| **Venous invasion (yes vs. no)** | 2.02 (0.98, 4.17) | 0.057 | 1.75 (0.86, 3.60) | 0.125 |
| **Perineural invasion (yes vs. no)** | 1.66 (0.85, 3.24) | 0.135 | 1.51 (0.78, 2.91) | 0.222 |
| **Any CTCs (yes vs. no)** | 2.02 (1.16, 3.51) | **0.013** | 2.49 (1.43, 4.33) | **0.001** |
| **Any CTCs at baseline (yes vs. no)** | 1.79 (0.97, 3.30) | 0.064 | 1.99 (1.10, 3.61) | **0.023** |
| **Any CTCs at 6 months (yes vs. no)** | 1.91 (0.59, 6.18) | 0.281 | 7.63 (2.60, 22.35) | **< 0.001** |
| **Any CTCs at 1 year (yes vs. no)** | 6.29 (2.49, 15.91) | **< 0.001** | 8.88 (3.25, 24.27) | **< 0.001** |
| **Any CTCs at 2 years (yes vs. no)** | 3.96 (1.25, 12.56) | **0.019** | 2.84 (0.94, 8.60) | 0.065 |
| **Any CTC increase (yes vs. no)** | 3.14 (1.56, 6.34) | **0.001** | 3.34 (1.67, 6.69) | **< 0.001** |
| **Any CTC decrease (yes vs. no)** | 1.59 (0.78, 3.25) | 0.201 | 2.20 (1.12, 4.32) | **0.022** |
| **CTC status (vs. none)**  Decrease + post-treatment CTCs  Decrease + baseline CTCs  No decrease + post-treatment CTCs  No decrease + baseline CTCs | 0.91 (0.12, 6.86)  1.54 (0.74, 3.21)  2.28 (1.02, 5.11)  8.40 (2.85, 24.75) | **0.002**  0.924  0.245  **0.046**  **< 0.001** | 2.34 (0.52, 10.41)  1.90 (0.94, 3.86)  2.68 (1.17, 6.10)  10.15 (3.34, 30.81) | **< 0.001**  0.266  0.076  **0.019**  **< 0.001** |

**HR** – Hazard ratio; **CI** – Confidence interval; **NR** – Not reported; **CRT** – Chemoradiotherapy;

**SUV_Max_** – Maximum standardized uptake values; **CTC** – Circulating tumour cells; P-values < 0.05 shown in **BOLD**;
