# Supplementary material for: The quality of life in neoadjuvant versus adjuvant therapy of esophageal cancer treatment trial (QUINTETT): Randomized parallel clinical superiority trial
Source: Thorac Cancer. 2022 May 24;13(13):1898–915. doi: 10.1111/1759-7714.14433 (PMC9250846; doi:10.1111/1759-7714.14433)
Supplement: Supplementary file 3 — Table S2 [file TCA-13-1898-s002.docx]

| **Supplemental Table 1. Summary of post-chemoradiotherapy adverse events with at least one grade ≥ 2 event for all patients and stratified by treatment arm (n=96).** | | | | | | | | | | | | | | | | | | | | |
| --- | --- | --- | --- | --- | --- | --- | --- | --- | --- | --- | --- | --- | --- | --- | --- | --- | --- | --- | --- | --- |
| **Adverse Event / Grade:** | **Arm #1: Neoadjuvant CRT** | | | | | | | | | **Arm #2: Adjuvant CRT** | | | | | | | | | **p-value** |  |
|  | **1** | **2** | | **3** | | **4** | | **5** | | **1** | **2** | | **3** | | **4** | | **5** | |  |  |
| **Abdominal pain** | 2 | | 2 | | 1 | | - | | - | - | | - | | - | | - | | - | **0.025** |  |
| **Acute coronary syndrome** | - | | - | | 2 | | - | | - | - | | - | | - | | 1 | | - | 0.237 |  |
| **Acute kidney injury** | - | | - | | - | | 1 | | - | - | | - | | - | | - | | - | 0.490 |  |
| **Anemia** | - | | - | | 3 | | - | | - | 1 | | 3 | | - | | - | | - | 0.059 |  |
| **Anxiety** | - | | 1 | | - | | - | | - | 1 | | 1 | | - | | - | | - | > 0.99 |  |
| **Arthritis** | - | | 1 | | - | | - | | - | - | | - | | - | | - | | - | 0.490 |  |
| **Aspiration** | - | | - | | - | | - | | - | - | | 1 | | - | | - | | - | > 0.99 |  |
| **Atrial fibrillation** | - | | - | | 2 | | - | | - | - | | - | | - | | - | | - | 0.237 |  |
| **Back pain** | - | | - | | - | | - | | - | - | | 1 | | - | | - | | - | > 0.99 |  |
| **Bone marrow hypocellular** | - | | - | | 3 | | - | | - | - | | - | | - | | - | | - | 0.114 |  |
| **Catheter related infection** | 1 | | 4 | | 1 | | - | | - | - | | 8 | | 1 | | - | | - | 0.675 |  |
| **Confusion** | - | | - | | 1 | | - | | - | - | | - | | - | | - | | - | 0.490 |  |
| **Constipation** | 3 | | 7 | | - | | - | | - | 6 | | 5 | | - | | - | | - | 0.626 |  |
| **Cough** | - | | - | | - | | - | | - | - | | 1 | | - | | - | | - | > 0.99 |  |
| **Dehydration** | 2 | | 7 | | 9 | | - | | - | 1 | | 2 | | 2 | | - | | - | **0.008** |  |
| **Depressed level of consciousness** | - | | - | | - | | 1 | | - | - | | - | | - | | - | | - | 0.490 |  |
| **Depression** | 1 | | - | | - | | - | | - | 2 | | 1 | | - | | - | | - | > 0.99 |  |
| **Diarrhea** | 3 | | 4 | | 2 | | - | | - | 6 | | 3 | | 1 | | - | | - | 0.780 |  |
| **Duodenal ulcer** | - | | 2 | | - | | - | | - | - | | - | | - | | - | | - | 0.237 |  |
| **Dyspepsia** | - | | - | | 1 | | - | | - | - | | - | | - | | - | | - | 0.490 |  |
| **Dysphagia** | 3 | | 2 | | 1 | | - | | - | 1 | | - | | - | | - | | - | 0.143 |  |
| **Enterocolitis infection** | - | | - | | - | | - | | - | - | | - | | - | | 2 | | - | 0.495 |  |
| **Epistaxis** | - | | - | | - | | - | | - | - | | 1 | | - | | - | | - | > 0.99 |  |
| **Esophageal obstruction** | - | | 1 | | 1 | | - | | - | - | | - | | - | | - | | - | 0.237 |  |
| **Esophagitis** | 2 | | 13 | | 6 | | - | | - | 1 | | 4 | | - | | - | | - | **< 0.001** |  |
| **Fatigue** | 7 | | 6 | | 2 | | - | | - | 5 | | 11 | | 1 | | - | | - | 0.589 |  |
| **Febrile neutropenia** | - | | - | | 12 | | - | | - | - | | - | | 1 | | 1 | | - | **< 0.001** |  |
| **Gastroesophageal reflux disease** | 2 | | 3 | | - | | - | | - | - | | 1 | | - | | - | | - | 0.172 |  |
| **Generalized muscle weakness** | - | | - | | - | | - | | - | - | | - | | 1 | | - | | - | > 0.99 |  |
| **Gum infection** | - | | 2 | | - | | - | | - | - | | - | | - | | - | | - | 0.237 |  |
| **Hearing impaired** | - | | 1 | | - | | - | | - | - | | - | | 1 | | - | | - | 0.742 |  |
| **Hip fracture** | - | | - | | 2 | | - | | - | - | | - | | - | | - | | - | 0.237 |  |
| **Hyperglycemia** | - | | - | | - | | 2 | | - | - | | 1 | | - | | - | | - | 0.237 |  |
| **Hyperkalemia** | - | | 1 | | - | | - | | - | - | | 1 | | - | | - | | - | > 0.99 |  |
| **Hypernatremia** | - | | - | | - | | 1 | | - | - | | - | | - | | - | | - | 0.490 |  |
| **Hypoglycemia** | - | | - | | - | | - | | - | - | | - | | 2 | | - | | - | 0.495 |  |
| **Hypokalemia** | 2 | | 1 | | - | | - | | - | - | | - | | 6 | | 1 | | - | **0.012** |  |
| **Hypomagnesemia** | - | | - | | - | | - | | - | - | | 2 | | 4 | | - | | - | **0.042** |  |
| **Hyponatremia** | 1 | | 1 | | - | | - | | - | - | | 2 | | - | | - | | - | 0.805 |  |
| **Hypotension** | 1 | | 1 | | - | | - | | - | 1 | | 1 | | - | | - | | - | > 0.99 |  |
| **Infusion site extravasation** | - | | - | | 1 | | - | | - | - | | - | | - | | - | | - | 0.490 |  |
| **Lip infection** | - | | - | | - | | - | | - | - | | 1 | | - | | - | | - | > 0.99 |  |
| **Lung infection** | - | | 2 | | - | | 1 | | - | - | | 5 | | 1 | | 1 | | - | 0.671 |  |
| **Mucositis (oral)** | - | | 9 | | 4 | | - | | - | 3 | | 5 | | 1 | | - | | - | 0.100 |  |
| **Nausea** | 9 | | 14 | | 6 | | - | | - | 7 | | 7 | | 10 | | - | | - | 0.197 |  |
| **Neutrophil count decreased** | 1 | | 2 | | 2 | | - | | - | 4 | | 2 | | 9 | | - | | - | 0.053 |  |
| **Non-cardiac chest pain** | 1 | | 1 | | - | | - | | - | - | | 1 | | - | | - | | - | 0.742 |  |
| **Palmar-plantar erythrodysesthes** | - | | - | | - | | - | | - | 2 | | 1 | | 2 | | - | | - | 0.147 |  |
| **Pericarditis** | - | | - | | 2 | | - | | - | - | | - | | - | | - | | - | 0.237 |  |
| **Peripheral motor neuropathy** | - | | - | | - | | - | | - | - | | 1 | | - | | - | | - | > 0.99 |  |
| **Peripheral sensory neuropathy** | - | | - | | - | | - | | - | 1 | | 4 | | - | | - | | - | 0.085 |  |
| **Pharyngitis** | - | | 5 | | - | | - | | - | - | | 3 | | - | | - | | - | 0.482 |  |
| **Phlebitis** | - | | 4 | | - | | - | | - | - | | - | | - | | - | | - | 0.054 |  |
| **Platelet count decreased** | 1 | | - | | 5 | | - | | - | - | | 2 | | 1 | | - | | - | 0.066 |  |
| **Pleural effusion** | - | | - | | - | | - | | - | - | | - | | 1 | | - | | - | > 0.99 |  |
| **Pneumonitis** | - | | - | | - | | - | | - | 1 | | 2 | | - | | - | | - | 0.495 |  |
| **Presyncope** | - | | 2 | | - | | - | | - | - | | 1 | | - | | - | | - | 0.613 |  |

**CRT** – Chemoradiotherapy; P-values < 0.05 shown as **BOLD**

| **Supplemental Table 1 (Continued). Summary of post-chemoradiotherapy adverse events with at least one grade ≥ 2 event for all patients and stratified by treatment arm (n=96).** | | | | | | | | | | | |
| --- | --- | --- | --- | --- | --- | --- | --- | --- | --- | --- | --- |
| **Adverse Event / Grade:** | **Arm #1: Neoadjuvant CRT** | | | | | **Arm #2: Adjuvant CRT** | | | | | **p-value** |
|  | **1** | **2** | **3** | **4** | **5** | **1** | **2** | **3** | **4** | **5** |  |
| **Pruritus** | - | 1 | - | - | - | 2 | - | - | - | - | 0.495 |
| **Rash (maculo-papular)** | 1 | 1 | - | - | - | 1 | 1 | - | - | - | > 0.99 |
| **Respiratory failure** | - | - | - | - | - | - | - | - | 1 | - | > 0.99 |
| **Sepsis** | - | - | - | 1 | - | - | - | - | - | - | 0.490 |
| **Sinusitis** | - | 1 | - | - | - | - | - | - | - | - | 0.490 |
| **Skin infection** | - | - | - | - | - | - | 1 | - | - | - | > 0.99 |
| **Superficial thrombophlebitis** | - | 3 | - | - | - | - | - | - | - | - | 0.114 |
| **Supraventricular tachycardia** | - | - | - | - | - | - | 1 | - | - | - | > 0.99 |
| **Syncope** | - | - | 4 | - | - | - | - | 1 | - | - | 0.199 |
| **Thromboembolic event** | - | 4 | 4 | - | 1 | - | 5 | 2 | 1 | - | 0.721 |
| **Tinnitus** | 1 | - | - | - | - | - | 2 | - | - | - | 0.495 |
| **Urinary tract infection** | - | 1 | - | - | - | - | - | - | - | - | 0.490 |
| **Vomiting** | 2 | 8 | 5 | - | - | 2 | 3 | 6 | - | - | 0.418 |
| **Weight loss** | 4 | 4 | 3 | - | - | 1 | 6 | 5 | 2 | - | 0.374 |
| **Wound infection** | - | 1 | - | - | - | - | 1 | - | - | - | > 0.99 |

**CRT** – Chemoradiotherapy; P-values < 0.05 shown as **BOLD**
