# Supplementary material for: The quality of life in neoadjuvant versus adjuvant therapy of esophageal cancer treatment trial (QUINTETT): Randomized parallel clinical superiority trial
Source: Thorac Cancer. 2022 May 24;13(13):1898–915. doi: 10.1111/1759-7714.14433 (PMC9250846; doi:10.1111/1759-7714.14433)
Supplement: Supplementary file 4 — Table S3 [file TCA-13-1898-s004.docx]

| **Supplemental Table 2. Summary of post-surgery adverse events with at least one grade ≥ 2 event for all patients and stratified by treatment arm (n=96).** | | | | | | | | | | | |
| --- | --- | --- | --- | --- | --- | --- | --- | --- | --- | --- | --- |
| **Adverse Event / Grade:** | **Arm #1: Neoadjuvant CRT** | | | | | **Arm #2: Adjuvant CRT** | | | | | **p-value** |
|  | **1** | **2** | **3** | **4** | **5** | **1** | **2** | **3** | **4** | **5** |  |
| **Abdominal pain** | - | 1 | - | - | - | - | 1 | - | - | - | > 0.99 |
| **Acute coronary syndrome** | - | - | 1 | - | - | - | - | - | - | - | 0.490 |
| **Acute kidney injury** | - | - | - | - | - | - | 1 | - | - | - | > 0.99 |
| **Agitation** | - | - | - | - | - | - | 1 | - | - | - | > 0.99 |
| **Allergic reaction** | - | - | - | - | - | - | - | 1 | - | - | > 0.99 |
| **Aspiration** | - | - | - | 2 | - | - | 2 | - | 1 | - | 0.617 |
| **Atelectasis** | - | - | 3 | - | - | - | - | - | - | - | 0.114 |
| **Atrial fibrillation** | 1 | 3 | 1 | - | - | - | 9 | - | - | - | 0.121 |
| **Back pain** | - | 2 | - | - | - | 1 | - | - | - | - | 0.237 |
| **Blocked feeding tube (replaced)** | - | - | 1 | - | - | - | - | 3 | - | - | 0.617 |
| **Bronchial obstruction** | - | - | - | - | - | - | 1 | - | - | - | > 0.99 |
| **Catheter related infection** | - | - | - | - | - | 2 | 4 | - | - | - | **0.042** |
| **Cholecystitis** | - | - | - | - | - | - | - | - | 1 | - | > 0.99 |
| **Chylothorax** | - | 4 | 2 | - | - | - | 1 | 3 | - | - | 0.385 |
| **Confusion** | - | - | - | - | - | - | 3 | - | - | - | 0.242 |
| **Constipation** | - | 1 | - | - | - | 1 | 1 | - | - | - | > 0.99 |
| **Dehydration** | - | - | - | - | - | - | 1 | - | - | - | > 0.99 |
| **Depression** | 1 | - | - | - | - | 1 | 1 | - | - | - | > 0.99 |
| **Diarrhea** | 3 | - | - | - | - | 3 | 1 | - | - | - | > 0.99 |
| **Diverticulitis / dumping syndrome** | - | 3 | - | - | - | - | - | - | - | - | 0.114 |
| **Dumping syndome** | 1 | 1 | - | - | - | 2 | - | - | - | - | 0.805 |
| **Duodenal perforation** | - | - | - | - | - | - | - | - | 1 | - | > 0.99 |
| **Duodenal ulcer** | - | - | - | - | - | - | - | - | 1 | - | > 0.99 |
| **Dysphagia** | 1 | 3 | - | - | - | 3 | 2 | 4 | - | - | 0.186 |
| **Dyspnea** | - | - | 1 | - | - | - | 1 | - | - | - | 0.742 |
| **Edema (limbs)** | - | - | - | - | - | - | 1 | - | - | - | > 0.99 |
| **Enterocolitis infection** | - | - | 1 | - | - | - | - | - | - | - | 0.490 |
| **Epistaxis** | - | - | - | - | - | - | 1 | - | - | - | > 0.99 |
| **Esophageal stent migration** | - | - | - | - | - | - | - | 2 | - | - | 0.495 |
| **Esophageal anastomotic leak** | 1 | 2 | 1 | 3 | - | - | 4 | 1 | 3 | - | 0.947 |
| **Esophageal fistula** | - | - | 1 | - | - | - | - | 1 | - | - | > 0.99 |
| **Esophageal obstruction** | - | - | 1 | - | - | - | - | 4 | - | - | 0.362 |
| **Esophageal stenosis** | - | - | 7 | - | - | - | - | 16 | 4 | - | **0.007** |
| **Esophageal stent migration requiring intervention** | - | - | - | - | - | - | - | - | 1 | - | > 0.99 |
| **Failure to thrive requiring admission** | - | - | - | 3 | - | - | - | - | - | - | 0.114 |
| **Fatigue** | 1 | - | - | - | - | 1 | 1 | - | - | - | > 0.99 |
| **Foot drop / nerve compression** | - | - | - | - | - | - | 2 | - | - | - | 0.495 |
| **Gastric anastomotic leak** | - | - | - | - | - | - | - | - | 1 | - | > 0.99 |
| **Gastric necrosis** | - | - | - | 2 | - | - | - | - | 1 | - | 0.613 |
| **Gastric perforation** | - | - | - | - | - | - | 1 | - | - | - | > 0.99 |
| **Gastric stenosis** | - | - | - | - | - | - | - | 2 | - | - | 0.495 |
| **Gastric ulcer** | - | - | - | - | - | - | 1 | - | - | - | > 0.99 |
| **Gastritis** | - | 1 | - | - | - | - | 1 | - | - | - | > 0.99 |
| **Gastroesophageal reflux disease** | 1 | 9 | - | - | - | 2 | 1 | - | - | - | **0.009** |
| **Gastroparesis** | 1 | 3 | 3 | - | - | 1 | 5 | 5 | - | - | 0.830 |
| **Granulation tissue (treated with silver nitrate)** | - | - | - | - | - | - | 1 | - | - | - | > 0.99 |
| **Hematoma** | 1 | - | - | - | - | - | - | 2 | - | - | 0.495 |
| **Horner’s syndrome (idiopathic)** | - | 1 | - | - | - | - | - | - | - | - | 0.490 |
| **Hypoglycemia** | - | 1 | - | - | - | - | - | - | - | - | 0.490 |
| **Hypotension** | - | - | - | - | - | - | - | 2 | - | - | 0.495 |
| **Ileus** | - | 1 | - | - | - | - | 1 | - | - | - | > 0.99 |
| **Intraoperative splenic injury** | - | - | - | - | - | - | 1 | - | - | - | > 0.99 |
| **Left ventricular systolic dysfunction** | - | - | 1 | - | - | - | - | - | - | - | 0.490 |
| **Lung infection** | - | 2 | 4 | 1 | - | - | 1 | - | - | - | **0.037** |
| **Mobitz type II atrioventricular block** | - | - | - | - | - | - | - | 1 | - | - | > 0.99 |
| **Myocardial infarction** | - | - | - | - | - | - | 1 | - | - | - | > 0.99 |
| **Nausea** | 1 | - | - | - | - | 2 | 1 | 1 | - | - | > 0.99 |

**CRT** – Chemoradiotherapy; P-values < 0.05 shown as **BOLD**

| **Supplemental Table 2 (Continued). Summary of post-surgery adverse events with at least one grade ≥ 2 event for all patients and stratified by treatment arm (n=96).** | | | | | | | | | | | |
| --- | --- | --- | --- | --- | --- | --- | --- | --- | --- | --- | --- |
| **Adverse Event / Grade:** | **Arm #1: Neoadjuvant CRT** | | | | | **Arm #2: Adjuvant CRT** | | | | | **p-value** |
|  | **1** | **2** | **3** | **4** | **5** | **1** | **2** | **3** | **4** | **5** |  |
| **Peripheral motor neuropathy** | - | - | - | - | - | - | 1 | - | - | - | > 0.99 |
| **Peripheral sensory neuropathy** | - | 1 | - | - | - | - | - | - | - | - | 0.490 |
| **Pleural effusion** | - | 1 | 2 | - | - | - | - | 2 | - | - | 0.805 |
| **Pleural infection** | - | - | 1 | - | - | - | - | - | - | - | 0.490 |
| **Pneumothorax** | - | 1 | 2 | - | - | 1 | - | - | - | - | 0.173 |
| **Postoperative hemorrhage** | - | - | - | - | - | - | - | 1 | - | - | > 0.99 |
| **Recurrent laryngeal nerve palsy** | 1 | 6 | 2 | - | - | 1 | 5 | 3 | 1 | - | > 0.99 |
| **Renal calculi** | - | 1 | - | - | - | - | - | - | - | - | 0.490 |
| **Respiratory failure** | - | - | - | 4 | - | - | - | - | 5 | - | > 0.99 |
| **Sepsis** | - | - | - | - | - | - | - | - | 1 | 1 | > 0.99 |
| **Skin ulceration** | - | - | - | - | - | 2 | - | - | 1 | - | 0.495 |
| **Stroke** | - | - | - | - | - | - | - | - | - | 2 | 0.495 |
| **Supraventricular tachycardia** | - | 2 | 1 | - | - | - | - | 1 | - | - | 0.484 |
| **Thromboembolic event** | - | 1 | 3 | - | - | - | - | - | - | - | 0.054 |
| **Transient ischemia attack** | - | 1 | - | - | - | - | 1 | - | - | - | > 0.99 |
| **Urinary retention** | - | 2 | - | - | - | - | 4 | - | - | - | 0.678 |
| **Urinary tract infection** | - | 1 | - | - | - | - | 5 | - | - | - | 0.204 |
| **Vomiting** | 1 | - | - | - | - | 5 | 2 | - | - | - | 0.090 |
| **Weight loss** | - | 2 | - | - | - | 3 | 5 | 2 | - | - | 0.065 |
| **Wound complication** | - | - | 1 | - | - | 1 | - | 5 | - | - | 0.155 |
| **Wound dehiscence** | - | 2 | 1 | - | - | 1 | 2 | 4 | - | - | 0.405 |
| **Wound infection** | - | 3 | 8 | - | - | - | 4 | 4 | 1 | - | 0.448 |
| **Subcutaneous emphysema** | - | - | - | - | - | - | 1 | - | - | - | > 0.99 |

**CRT** – Chemoradiotherapy; P-values < 0.05 shown as **BOLD**
